# Elective nodal irradiation of high-risk regions is superior to involved field radiotherapy for limited-stage small cell lung cancer: a propensity score-matched retrospective study

**DOI:** 10.1186/s13014-026-02845-6

**Published:** 2026-04-28

**Authors:** Zheng Zhang, Meng Yan, Jiakun Gao, Gengmin Niu, Yihan Guo, Boyu Qian, Ming Li, Kai Ren, Xue Li, Lujun Zhao

**Affiliations:** 1 Department of Radiation Oncology, Tianjin’s Clinical Research Center for Cancer, Key Laboratory of Cancer Prevention and Therapy, Tianjin Medical University Cancer Institute & Hospital, National Clinical Research Center for Cancer, Tianjin, China; 2 Public Laboratory, Tianjin’s Clinical Research Center for Cancer, Key Laboratory of Cancer Prevention and Therapy, Tianjin Medical University Cancer Institute & Hospital, National Clinical Research Center for Cancer, Tianjin, China

**Keywords:** Elective nodal irradiation, Involved field radiotherapy, Limited-stage small cell lung cancer, Immunotherapy

## Abstract

**Background and purpose:**

Whether to omit elective nodal irradiation (ENI) in thoracic radiotherapy for limited-stage small cell lung cancer (LS-SCLC) remains controversial. The purpose of this study was to compare the efficacy and safety of ENI in high-risk lymph node regions compared to involved field radiotherapy (IFRT).

**Materials and methods:**

We conducted a real-world retrospective study of LS-SCLC who received radical simultaneous dose-reduction radiotherapy (SDR-RT) and chemotherapy with or without immunotherapy from January 2018 to June 2023. Patients were divided into two groups based on the target volume delineation method: the ENI group and the IFRT group. Propensity score matching (PSM) was applied to balance the observable potential confounding factors between the two groups. The primary endpoints were overall survival (OS).

**Results:**

A total of 540 eligible patients were enrolled, of whom 196 (36.3%) received ENI and 344 (63.7%) received IFRT. After PSM, the clinical characteristics of both groups were balanced, including 183 patients each. The median OS was 26.7 months in the IFRT group versus 37.7 months in the ENI group (*P* = 0.007). The median PFS was 8.7 months in the IFRT group versus 12.2 months in the ENI group (*P* = 0.006). The median DMFS was 12.3 months and 28.6 months in the two groups (*P* = 0.004), respectively. The incidence of radiation pneumonia (*P* = 0.646) and esophagitis (*P* = 0.493) were similar between the two groups. The 1-year cumulative incidence rate of ENF was 13.9% (95% CI 8.8–18.9%) in the IFRT group and 2.7% (0.4–5.1%) in the ENI group (Gray’s *p* < 0.001).

**Conclusion:**

ENI did not significantly increase the rate of adverse reactions compared to IFRT, while effectively prolonging OS, PFS, and DMFS, and reducing the risk of ENF in patients with LS-SCLC.

**Clinical trial number:**

Not applicable.

**Supplementary Information:**

The online version contains supplementary material available at 10.1186/s13014-026-02845-6.

## Introduction

Thoracic radiotherapy (TRT) is one of the standard treatments for limited-stage small cell lung cancer (LS-SCLC). Despite the high sensitivity of LS-SCLC to radiotherapy, the prognosis is still poor, with a 3-year overall survival of approximately 56.5% [1]. Although involved field radiotherapy (IFRT) has been widely recommended to limit toxicity in TRT of LS-SCLC [2–4], the recommendation is not absolute [5, 6]. For example, inclusion of the ipsilateral supraclavicular region in cases of upper mediastinal (station 2/3A) involvement without pretreatment positron emission tomography (PET), or selective treatment of the ipsilateral hilum or supraclavicular nodal or subcarinal regions in some trials [5–8]. These exceptions underscore the uncertainty of omitting elective nodal irradiation (ENI) in high-risk lymph node regions where subclinical lesions may exist.

Advances in imaging and radiation have changed the treatment landscape. PET-CT improves accuracy of detecting subtle small lesions and supports the rationale for IFRT [3, 9, 10]. However, relevant pathological evidence and meta-analyses underscore the non-negligible risk of occult nodal metastases in PET-CT negative patients [11–13]. Meanwhile, the use of modulated techniques enhances conformability and reduces the incidental dose to uninvolved nodal areas compared with 3-dimensional conformal treatment [4, 14]. Consequently, the decrease of incidental dose may lead to subclinical disease unsubstantially treated, making conclusions [4, 15] from 3-dimensional conformal treatment need to be evaluated. The recently completed SDR-RT trial [8] has demonstrated that simultaneous integrated dose reduction radiotherapy (SDR-RT), while maintaining a definitive radiation dose to the gross tumor volume (GTV), can relatively reduce the irradiation dose to the surrounding subclinical region (clinical target volume, CTV). Compared with conventional fractionated radiation therapy, this approach achieves comparable survival outcomes while reducing normal tissue toxicity and allowing for a safe expansion of the treatment field. Additionally, consolidation immunotherapy after chemoradiotherapy, as demonstrated in the ADRIATIC study [16], has changed treatment paradigms and may influence patterns of failure. And the outcomes of the first stage in adebrelimab with concurrent chemoradiation (cCRT) in LS-SCLC demonstrated that initiating immunotherapy from the induction phase is both feasible and effective [17, 18]. All in all, these advances in imaging, radiotherapy and systemic therapy have created a different clinical context and introduce new uncertainties in the necessity of ENI and the appropriate prophylactic dose required to control disease while minimizing toxicity.

Therefore, this study aims to evaluate whether selective ENI improves survival or regional control compared with IFRT in LS-SCLC treated with modulated techniques, particularly treated with immunotherapy.

## Materials and methods

### Study population and eligibility criteria

This study retrospectively included 540 LS-SCLC patients who received definitive chemoradiotherapy at Tianjin Medical University Cancer Hospital and Tianjin Cancer Hospital Airport Hospital from January 2018 to June 2023. The inclusion criteria were as follows: (1) pathologically confirmed SCLC; (2) age ≥ 18 years old; (3) LS-SCLC (stages I-IIIC) as defined by the American Joint Committee on Cancer (AJCC), 8th edition of tumor lymph node metastasis (TNM stage); (4) completion of chest computed tomography (CT), cervical and abdominal ultrasound, brain magnetic resonance imaging (MRI), and bone scan imaging, or alternatively, PET and brain MRI prior to radiotherapy; (5) receipt of definitive chemoradiotherapy. (6) Eastern Cooperative Oncology Group (ECOG) performance status is 0 to 2. The exclusion criteria: (1) concurrent second primary tumor; (2) incomplete case data or missing main clinical information; (3) underwent pneumonectomy. This study was approved and approved by the Ethics Committee of Tianjin Medical University Cancer Hospital (approval number: bc20254192), and signing of informed consent forms was waived for retrospective research.

### Chemotherapy

All patients received induction chemotherapy, and some patients received concurrent or adjuvant chemotherapy. The chemotherapy regimen consisted of etoposide and platinum-based drugs (cisplatin or carboplatin). Etoposide was administered intravenously at a 100 mg/m2 on days 1–3 of each cycle. Cisplatin (75 mg/m2, day 1) or carboplatin (AUC 5, on day 1) was administered intravenously. These regimens were repeated every three weeks. Immunotherapy regimens involved in this study included anti-PD-L1 (atezolizumab and durvalumab) and PD-1 antibodies (sintilimab, tislelizumab, camrelizumab, and serplulimab), and immunotherapy was started concurrently with induction chemotherapy, with the continuation of immunotherapy after the radiotherapy at the discretion of the treating physician and the patient for a maximum of 24 months.

### Radiotherapy planning and target volume definition

This study compared two groups of patients treated with different target volume concepts: IFRT versus ENI. All patients were scanned using a large-bore spiral CT simulator. The slice thickness is 2.5–5 mm. All patients received intensity-modulated radiotherapy (IMRT) or volumetric modulated arc therapy (VMAT) and received simultaneous integrated dose reduction (SDR) radiotherapy (SDR-RT) [19, 20]. Previous studies on the basic anatomical lymphatic drainage pathways of lung cancer have revealed that the pattern of micrometastasis based on the primary lobe is generally consistent with that of overt lymph node involvement [21]. The pattern of lymph node metastasis typically involves initial passage through the hilar lymph nodes. For tumors originating in the right upper lobe, the right paratracheal lymph nodes are the most common site of mediastinal metastasis. The subcarinal lymph nodes are more frequently involved in malignancies of the right middle and lower lobes. Tumors in the left upper lobe tend to drain to the aortopulmonary window and periaortic lymph nodes. Similar to the right lower lobe, tumors in the left lower lobe most often drain to the subcarinal lymph nodes [21–23]. Additional studies have shown that pre-chemotherapy mediastinal lymph node metastasis in stations 2 and 3 is a risk factor for supraclavicular recurrence after treatment of LS-SCLC [24, 25]. Therefore, in this study, the ENI region was primarily defined based on drainage pathways observed in established anatomical knowledge, targeting downstream high-risk lymph node regions along the ipsilateral hilar, station 4 L/R, and station 2 L/R to station 1 L/R pathways for ENI. Additionally, for patients with primary tumors of the left upper lobe, the ENI range can extend to cover stations 5/6. For patients with middle/lower lobe tumors and hilar involvement, the range can extend to cover station 7. Based on the International Association for the Study of Lung Cancer (IASLC) consensus lymph node map [26] and the basic anatomical lymph node drainage and micrometastasis patterns of lung cancer [21, 22], elective irradiation was performed by extending CTV to at least one complete station of high-risk uninvolved lymph node stations adjacent to the primary lesion or mediastinal involved lymph node station. The gross tumor volume (GTV) comprised the primary tumor (GTV-T) and lymph nodes identified as positive prior to chemotherapy (GTV-N). Lymph nodes were considered positive if they met any of the following criteria: a positive tumor cell biopsy result; a short-axis diameter of ≥ 1 cm in the mediastinum at initial staging; an F-18 fluorodeoxyglucose standard uptake value of ≥ 2.5 on PET scan; or the presence of small nodal clusters in a region with a short diameter < 1 cm. Additionally, lymph nodes exhibiting significant changes following induction systemic therapy were also classified as positive. Planned gross tumor volume (PTVG), clinical target volume (CTV) and planned target volume (PTV) according to Chen’s definitions [4]. For the IFRT group, the clinical target volume (CTV) included the clinical target volume-tumor (CTV-T) and the clinical target volume-lymph node (CTV-N). CTV-T included the postchemotherapy residual GTV-T with a margin of 0.5 cm. CTV-N contained the positive prechemotherapy lymph node regions. The lymph node regions were also included in the radiation fields as CTV-N even if the lymph node disappeared after induction chemotherapy. For the ENI group, CTV included CTV-T and CTV-N and high-risk selective uninvolved lymph node regions (Figure S1, Figure S2). The CTV was subsequently edited based on anatomy. The 0.5 cm expansion to GTV was defined as the planning gross tumor volume (PTVG). The 0.5 cm expansion to CTV was defined as the planning target volume (PTV).

The prescribed radiation dose for PTVG is 60 Gy with a division of 2.0 Gy/day, and the prescribed radiation dose for PTV is 54 Gy with a division of 1.8 Gy/day, 5 fractions per week. The isodose line of 60 Gy covered at least 95% of the PGTV, and the isodose line of 54 Gy covered at least 95% of the PTV. Dose constraints for the organs at risk (OARs) were set as follows: mean heart dose ≤ 20 Gy; heart V30 (volume percentage of the heart receiving radiation doses ≥ 30 Gy) ≤ 40%; and heart V40 ≤ 30%; the maximal dose to spinal cord ≤ 45 Gy; mean esophageal dose < 34 Gy and the maximal dose to esophageal ≤ 60 Gy; mean lung dose ≤ 20 Gy and lung V20 (volume percentage of the heart receiving radiation doses ≥ 20 Gy) ≤ 30%. The dose constraints of the OARs should be kept “as low as reasonably achievable” (ALARA principle). The radiotherapy target volumes for all enrolled patients were delineated by two experienced radiation oncologists from the same medical center, with treatment plans generated by a medical physicist. All plans were jointly reviewed and evaluated by the team, in accordance with the aforementioned guidelines for target volume delineation, prescription dose, and dose constraints.

### Clinical endpoints

The primary clinical endpoints of the study were overall survival (OS) and ENF, while the secondary endpoints included progression-free survival (PFS), locoregional recurrence-free survival (LRFS), and distant metastasis-free survival (DMFS). Overall survival (OS) was defined as the interval from the start date of radiotherapy to the date of death from any cause or the last follow-up. Progression-free survival (PFS) was determined from the start of radiation therapy to the date of progression, death from any cause, or the last follow-up, whichever occurs first. Distant metastasis-free survival (DMFS) was defined as the time from the commencement of radiotherapy to the occurrence of distant metastasis or death from any cause. Locoregional recurrence-free survival (LRFS) was calculated from the initiation of radiation therapy to the date of locoregional recurrenc*e* or death from any cause. The locoregional recurrence (LR) was defined as the first instance of tumor recurrence occurring either at the primary site or within the lymph nodes along its natural lymphatic drainage pathway (including the supraclavicular fossa), with pleural effusion and distinct tumor nodules in a different ipsilateral lobe or the contralateral lung excluded. Elective nodal failure (ENF) was defined following Rosenzweig’s definition [27] as a recurrence in any lymph node region that was initially uninvolved in the absence of local failure. If the patient had distant metastatic disease as the first site of failure without locoregional recurrence, failure in any originally uninvolved lymph node region was considered an ENF. Involved-field nodal failure (IFNF) was defined as a nodal failure in all involved lymph node stations. Isolated ENF was defined by the absence of recurrence in both the primary tumor failure or IFNF. Additionally, we assessed adverse reactions, particularly radiation pneumonitis (RP) and radiation esophagitis (RE), in accordance with the Common Terminology Criteria for Adverse Events (CTCAE), version 5.0.

### Statistical analysis

Continuous variables in patient characteristics were evaluated by the Mann-Whitney test, while categorical variables were evaluated by the Chi-square test or Fisher’s exact test. OS, DMFS, LRFS and PFS were estimated using the Kaplan-Meier method, and the log-rank test was used to assess intergroup differences. Prognostic analysis for DMFS, LRFS, and OS was conducted using both univariate and multivariate Cox proportional hazards regression models. Variables that showed a P value < 0.1 in the univariate analysis were included in the subsequent multivariate Cox regression to assess their association with potential predictors. To further mitigate confounding effects, one-to-one propensity score matching (PSM) with a caliper width of 0.2 standard deviations was implemented. Propensity scores were calculated based on age, gender, T stage, N stage, clinical stage, GTV volume, supraclavicular lymph node metastasis, therapy combined with immune checkpoint inhibitors (ICIs), ECOG performance status, tumor location, concurrent chemoradiotherapy, radiotherapy modality, the timing of thoracic radiotherapy, total chemotherapy cycles, and auxiliary PET staging. Covariates were defined as: gender classified as male or female; age classified as ≤ 60 years or > 60 years; T stage classified as T1, T2, T3, and T4; N stage classified as N1, N2, N3, and N4; TNM stage classified as Ⅰ-Ⅱ, ⅢA, ⅢB, and ⅢC; supraclavicular lymph node (SCLN) metastasis classified as none, unilateral, or bilateral; GTV volume classified according to the median value as > 46 cc or ≤ 46 cc; tumor location classified as central or peripheral (tumors were classified as central if primary tumor involved the segmental or more proximal bronchi, and peripheral if primary tumor was located distal to the subsegmental bronchi); chemotherapy cycles classified as 2–4, 5–6, or more than 6 cycles; and the timing of thoracic radiotherapy (TRT) classified as early (TRT initiated after 1–2 cycles of chemotherapy) or late (TRT initiated after 3 or more cycles of chemotherapy). To assess the robustness of the results, two sensitivity analysis methods were used to control for confounding: multivariate Cox regression adjusted for the inverse probability of treatment weighting (IPTW), and standardized mortality ratio (SMR) weighting. OS and PFS subgroup analyses were performed to evaluate the consistency of treatment effects among patient subgroups. An unstratified Cox proportional hazards model was employed for subgroup analyses, with treatment included as a covariate. Cumulative incidence curves were used to estimate the cumulative risk of ENF over time, and Gray’s test was used to compare the cumulative incidence of ENF between the two groups. In the analysis, death or primary tumor failure or IFNF without evidence of ENF was considered competing risk in the analysis. Statistical significance was set at *P* < 0.05, and all tests were two-sided. All statistical analyses were performed using SPSS version 27 (IBM, Armonk, NY, USA) and R version 4.5.1.

## Results

### Patients characteristics

A total of 540 patients were included in this study, patients were divided into two groups based on the target volume delineation method, 196 (36.3%) received ENI and 344 (63.7%) received IFRT. Detailed clinical characteristics are shown in Table S1. Compared to the IFRT group, the ENI group had more peripheral types (*P* = 0.031) and more utilization of VMAT (*P* < 0.001). After PSM, the clinical characteristics of both groups were balanced, with 183 patients included in each group (Table 1).

**Table 1:** Baseline demographic and clinical characteristics of the patients

Characteristic	IFRT (*N* = 183)	ENI (*N* = 183)	*P*-value	SMD
Gender, n (%)			0.390	
Female	40 (21.86)	47 (25.68)		0.088
Male	143 (78.14)	136 (74.32)		-0.088
Age, n (%)			0.914	
>60	71 (38.80)	70 (38.25)		-0.011
≤60	112 (61.20)	113 (61.75)		0.011
ECOG, n (%)			0.943	
0	37 (20.22)	36 (19.67)		-0.014
1	142 (77.60)	144 (78.69)		0.027
2	4 (2.19)	3 (1.64)		-0.043
T, n (%)			0.718	
1	29 (15.85)	33 (18.03)		0.057
2	72 (39.34)	64 (34.97)		-0.092
3	35 (19.13)	32 (17.49)		-0.043
4	47 (25.68)	54 (29.51)		0.084
N, n (%)			0.992	
0	6 (3.28)	5 (2.73)		-0.034
1	18 (9.84)	18 (9.84)		0.000
2	80 (43.72)	80 (43.72)		0.000
3	79 (43.17)	80 (43.72)		0.011
Clinical Stage, n (%)			0.996	
Ⅰ-Ⅱ	15 (8.20)	14 (7.65)		-0.021
ⅢA	49 (26.78)	50 (27.32)		0.012
ⅢB	85 (46.45)	84 (45.90)		-0.011
ⅢC	34 (18.58)	35 (19.13)		0.014
SCLN metastasis, n (%)			0.549	
No	155 (84.70)	159 (86.89)		0.065
Bilateral metastasis	0 (0.00)	0 (0.00)		
Unilateral metastasis	28 (15.30)	24 (13.11)		-0.065
Tumor type, n (%)			0.819	
Central	128 (69.95)	130 (71.04)		0.024
Peripheral	55 (30.05)	53 (28.96)		-0.024
Smoking, n (%)			1.000	
No	45 (24.59)	45 (24.59)		0.000
Yes	138 (75.41)	138 (75.41)		0.000
GTV volume (cc), n (%)			0.463	
>46	87 (47.54)	80 (43.72)		-0.077
≤46	96 (52.46)	103 (56.28)		0.077
PCI, n (%)			0.599	
No	149 (81.42)	145 (79.23)		-0.054
Yes	34 (18.58)	38 (20.77)		0.054
Radiotherapy modality, n (%)			0.599	
IMRT	105 (57.38)	100 (54.64)		-0.055
VMAT	78 (42.62)	83 (45.36)		0.055
CCRT, n (%)			0.833	
No	101 (55.19)	103 (56.28)		0.022
Yes	82 (44.81)	80 (43.72)		-0.022
Combined with ICIs, n (%)			0.874	
No	161 (87.98)	160 (87.43)		-0.016
Yes	22 (12.02)	23 (12.57)		0.016
TTRT, n (%)			0.403	
early TRT	96 (52.46)	88 (48.09)		-0.087
late TRT	87 (47.54)	95 (51.91)		0.087
Total chemotherapy cycles, n (%)			0.977	
2–4	101 (55.19)	99 (54.10)		-0.022
5–6	76 (41.53)	78 (42.62)		0.022
>6	6 (3.28)	6 (3.28)		0.000
PET CT, n (%)			0.753	
No	97 (53.01)	100 (54.64)		0.033
Yes	86 (46.99)	83 (45.36)		-0.033

Abbreviation: ECOG, Eastern Cooperative Oncology Group; SCLN, supraclavicular lymph node; PCI, Prophylactic Cranial Irradiation; CCRT, concurrent chemoradiotherapy; ICIs, immune checkpoint inhibitors; TTRT, the timing of thoracic radiotherapy. early TRT, thoracic radiotherapy initiated after 1–2 cycles of chemotherapy. late TRT, thoracic radiotherapy initiated after 3 or more cycles of chemotherapy; PET, positron emission tomography

### ENI VS IFRT survival analysis

Across the entire population, the median follow-up time was 42.0 months (range, 21.5–88.2). The median PFS and OS were 18.5 months and 23.2 months, respectively. During the study, 330 patients experienced progressive disease (PD), including 205 patients (59.6%) in the IFRT group and 125 patients (63.8%) in the ENI group. A total of 299 patients died, of which 200 patients (58.1%) and 99 patients (50.5%) in the two groups, respectively.

**Fig. 1:**
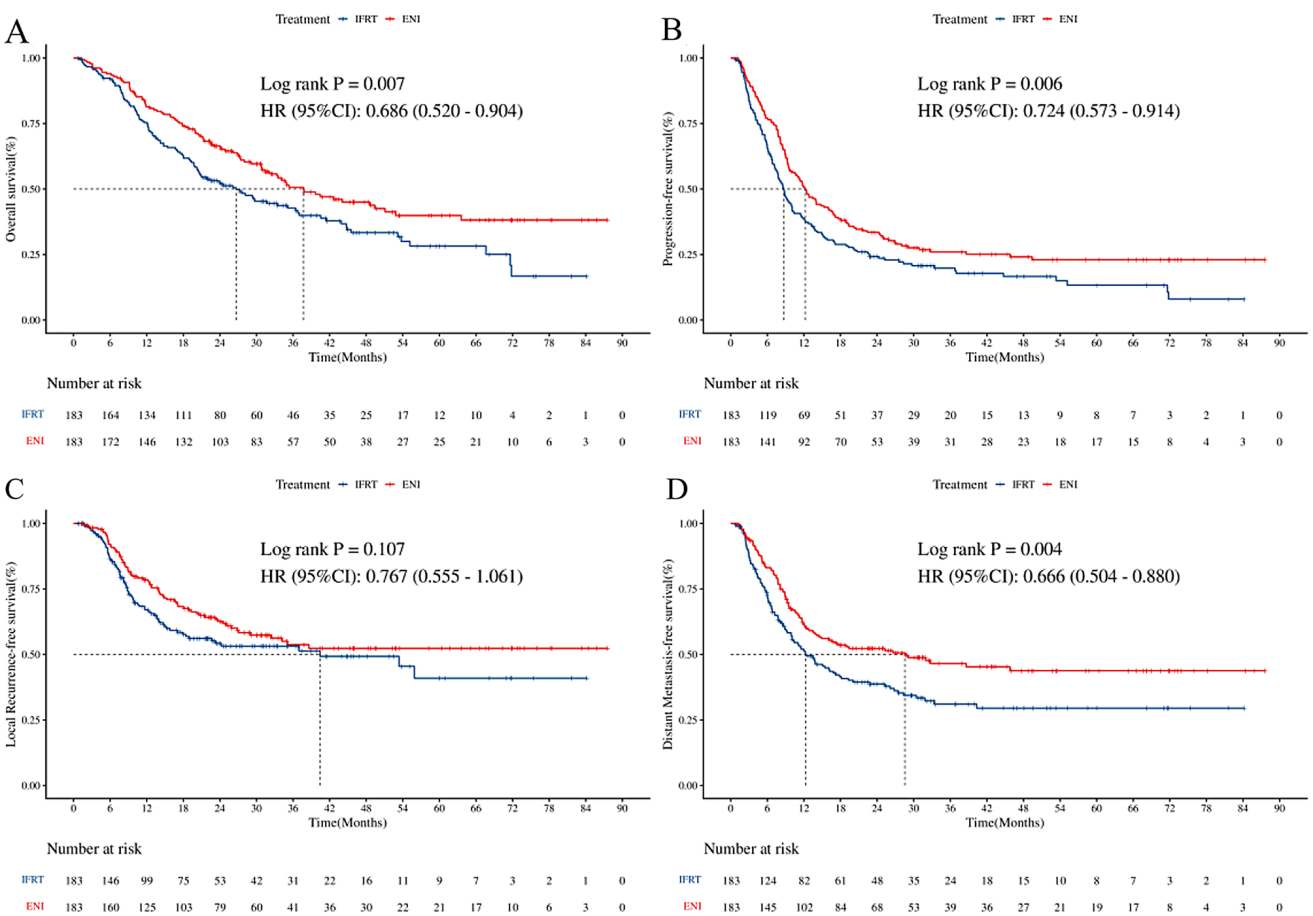
OS, PFS, DMFS and LRFS between the IFRT and ENI groups after PSM. (**A**) OS from the initiation of radiotherapy after PSM. (**B**) PFS from the initiation of radiotherapy after PSM. (**C**) DMFS from the initiation of radiotherapy after PSM. (**D**) LRFS from the initiation of radiotherapy after PSM. Abbreviation: OS overall survival, PFS progression-free survival, LRFS locoregional recurrence-free survival, IFRT Involved-field radiotherapy, DMFS distant metastasis-free survival; ENI, Elective Nodal Irradiation, PSM propensity score matching

ENI was associated with improved survival compared with IFRT as shown in Fig. 1. The median OS was 27.3 months in the IFRT group and 37.7 months in the ENI group (HR = 0.712, [95% CI: 0.559–0.906]; *P* = 0.006). The median DMFS was 15.4 months (95% CI: 12.17–23.90) in the IFRT group and 28.6 months (95% CI: 14.47-NA) in the ENI group (HR, 0.773, [95% CI: 0.604–0.990]; *P* = 0.040). No significant differences were observed in PFS (HR, 0.851, [95% CI: 0.695–1.043]; *P* = 0.120) or LRFS (HR, 0.938, [95% CI: 0.707–1.245]; *P* = 0.659). (Figure S3)

**Fig. 2:**
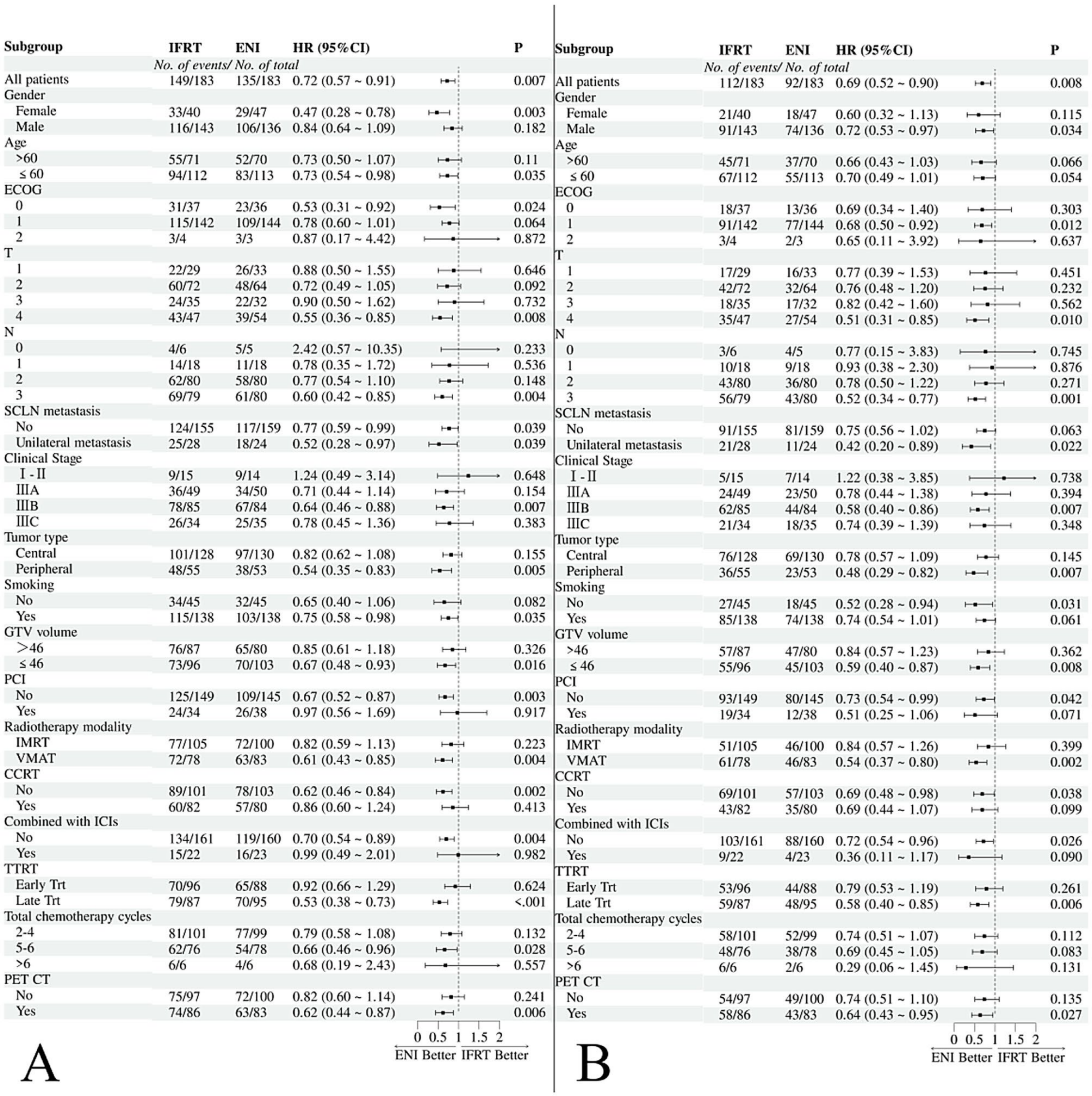
(**A**) Subgroup analyses of prognostic factors for PFS in the patients after PSM. (**B**) Subgroup analyses of prognostic factors for OS in the patients after PSM. Abbreviation: ECOG, Eastern Cooperative Oncology Group; SCLN, supraclavicular lymph node; PCI, Prophylactic Cranial Irradiation; CCRT, concurrent chemoradiotherapy; ICIs, immune checkpoint inhibitors; TTRT, the timing of thoracic radiotherapy. early TRT, thoracic radiotherapy initiated after 1–2 cycles of chemotherapy. late TRT, thoracic radiotherapy after 3 or more cycles of chemotherapy; PET, positron emission tomography

After 1:1 PSM, patients receiving ENI showed continued improvement in OS and DMFS. The median OS was 26.7 months (95% CI: 20.47–36.77) in the IFRT group and 37.7 months (95% CI: 31.40–52.83) in the ENI group (HR, 0.686,[95% CI: 0.520–0.904]; *P* = 0.007) (Fig. 1A). The median PFS was 8.7 months (95% CI: 7.63–10.07) in the IFRT group and 12.2 months (95% CI: 10.03–16.10) in the ENI group (HR, 0.724, [95% CI: 0.573–0.914]; *P* = 0.006) (Fig. 1B). The median LRFS was 40.4 months (95% CI: 18.43 - NA) in the IFRT group and was not reached in the ENI group (HR, 0.767,[95% CI: 0.555–1.061]; *P* = 0.209) (Fig. 1C). The median DMFS was 12.3 months (95% CI: 10.07–17.83) in the IFRT group and 28.6 months (95% CI: 14.47 - NA) in the ENI group (HR, 0.666,[95% CI: 0.504–0.880]; *P* = 0.004) (Fig. 1D). Multivariate analysis (Table S3) further confirmed the value of ENI on improving OS (HR = 0.69, *P* = 0.010), PFS (HR = 0.69, *P* = 0.002), and DMFS (HR = 0.68, *P* = 0.007). In addition, it was found that PCI significantly benefited OS, PFS, and DMFS. Male sex correlated with worse OS and PFS. The sensitivity analysis results are shown in Table S4. Further subgroup analysis showed that the OS and PFS benefit of ENI was observed in most of the pre-specified subgroups (Fig. 2).

### Radiotherapy-related toxicity

After PSM, radiotherapy toxicities were similar in both groups, where grade 3 or higher pneumonitis was reported in 5 (2.74%) of 183 patients in the IFRT group and 7 (3.83%) of 183 patients in the ENI group, grade 3 or higher esophagitis was reported in 7 (3.83%) of 183 patients in the IFRT group and 6 (3.28%) of 183 patients in the ENI group. Particularly, 1 patient died from pneumonitis in the IFRT group (Table 2). The dosimetric comparison results are shown in Table S5.

**Table 2:** Treatment-related toxicities of the IFRT group and ENI group

Characteristic	Before PSM			After PSM		
	IFRT (*n* = 344)	ENI (*n* = 196)	*P*-value	IFRT (*n* = 183)	ENI (*n* = 183)	*P*-value
Radiation esophagitis, n(%)			0.421			0.493
0–1	271 (78.78)	145 (73.98)		144 (78.69)	136 (74.32)	
2	63 (18.31)	45 (22.96)		32 (17.49)	41 (22.40)	
3	10 (2.91)	6 (3.06)		7 (3.83)	6 (3.28)	
4	0 (0.00)	0 (0.00)		0 (0.00)	0 (0.00)	
5	0 (0.00)	0 (0.00)		0 (0.00)	0 (0.00)	
Radiation pneumonitis, n(%)			0.550			0.646
0–1	289 (84.01)	162 (82.65)		155 (84.70)	151 (82.51)	
2	48 (13.95)	27 (13.78)		23 (12.57)	25 (13.66)	
3	6 (1.74)	7 (3.57)		4 (2.19)	7 (3.83)	
4	0 (0.00)	0 (0.00)		0 (0.00)	0 (0.00)	
5	1 (0.29)	0 (0.00)		1 (0.55)	0 (0.00)	

### Clinical failure patterns and the incidence of ENF

**Fig. 3:**
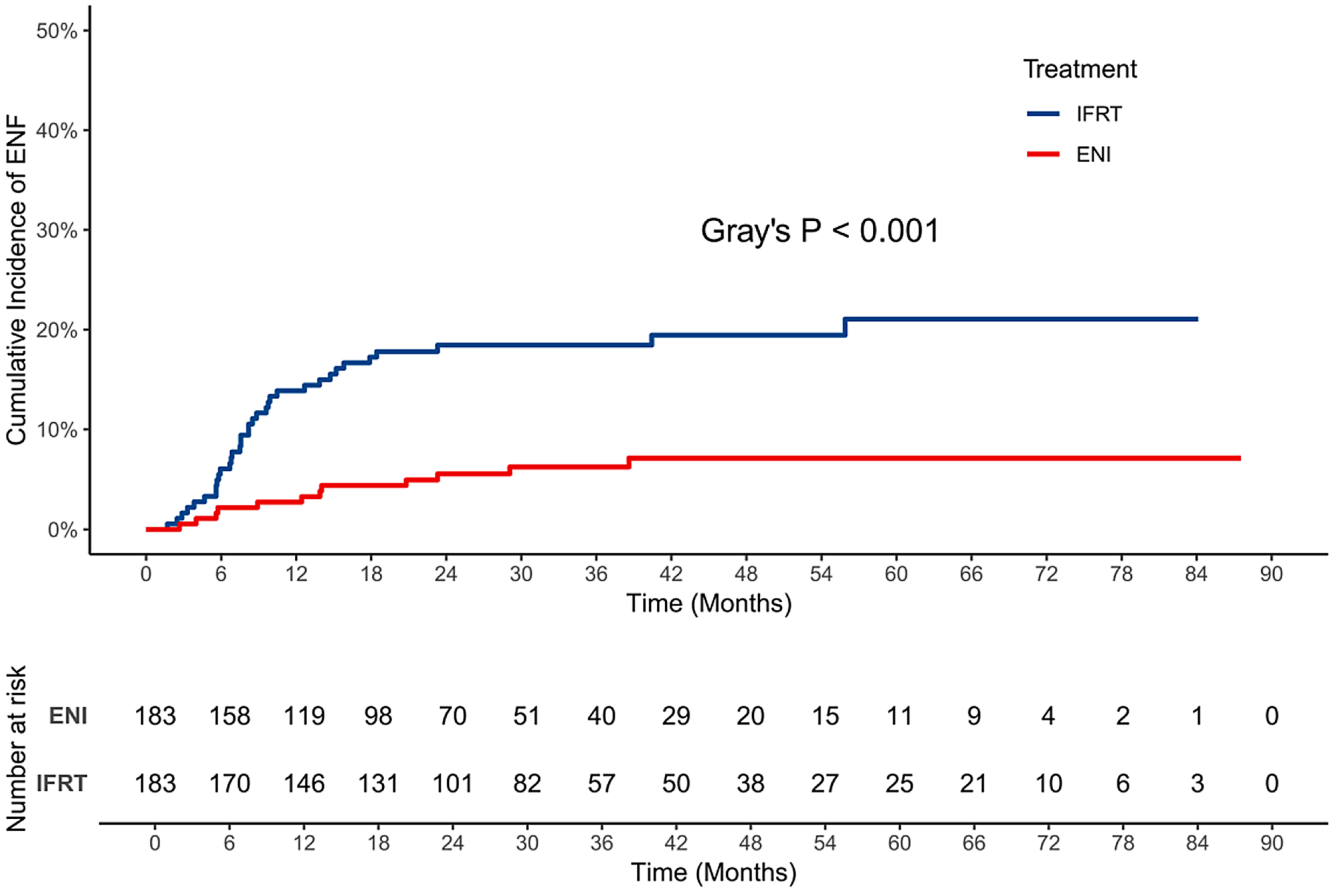
Cumulative incidence of ENF after PSM

In the total population, a total of 330 patients experienced locoregional recurrence and distant metastasis, a total of 58 patients developed ENF (Table S6, Table S7). The cumulative ENF rate was significantly higher in the IFRT group compared to the ENI group (Gray’s *p* = 0.0319). The 1-year, 3-year, and 5-year cumulative incidence rates of ENF in the IFRT and ENI group were 9.1% (95% CI 6-12.2%) vs. 3.6% (1-6.2%), 12.5% (8.9–16%) vs. 6.9% (3.2–10.5%), and 14.1% (9.9–18.3%) vs. 7.7% (3.7–11.6%), respectively. After PSM, the cumulative incidence of ENF remained significantly higher in the IFRT group compared to the ENI group, (Gray’s *p* < 0.001) (Fig. 3). The 1-year, 3-year, and 5-year cumulative incidence rates of ENF in the IFRT and ENI group were 13.9% (95% CI 8.8–18.9%) vs. 2.7% (0.4–5.1%), 18.4% (12.7–24.2%) vs. 6.3% (2.6–9.9%), and 21.1% (14.4–27.7%) vs. 7.1% (3.2–11.1%), respectively.

### ENI and immunotherapy

Multivariate analysis of the overall population confirmed the positive effect of the treatment that was combined with immunotherapy on improving OS (HR, 0.66; 95% CI: 0.45–0.98; *P* = 0.037) and DMFS (HR, 0.66; 95% CI: 0.45–0.96; *P* = 0.031) (Table S2). A total of 77 out of 540 patients used ICIs, including 53 patients in the IFRT group and 24 patients in the ENI group. In patients who received ICIs, compared with IFRT, ENI was also associated with improved OS, the median OS was not reached in both groups (HR, 0.379, [95% CI: 0.144–0.993]; *P* = 0.040), but no significant differences were seen in PFS (HR, 1.061, [95% CI: 0.592–1.901]; *P* = 0.843), DMFS (HR, 0.790, [95% CI: 0.363–1.717]; *P* = 0.550), and LRFS (HR, 1.627, [95% CI: 0.752–3.520]; *P* = 0.212). Among immunotherapy patients, 24 patients in the ENI group had no ENF, while 3 of 53 patients in the IFRT group had ENF.

## Discussion

In the study, we found that ENI in high-risk lymph node regions prolonged OS, PFS and DMFS in LS-SCLC, while not increasing adverse reactions compared with IFRT. Furthermore, we initially observed that using this ENI delineation method reduced the risk of ENF effectively. To our knowledge, this is the first study to assess the value of ENI under intensity modulated radiotherapy in LS-SCLC, and it included patients receiving immunotherapy, providing a novel optimized angle for thoracic radiotherapy in LS-SCLC.

The CONVERT and CALGB 30,610 trials [28–31] demonstrated that twice-daily hyperfractionated regimen (45 Gy in 30 fractions) and high-dose once-daily fractionated regimens (66–70 Gy in 33–35 fractions) yielded similar survival outcomes in patients with LS-SCLC. Nevertheless, high-dose once-daily fractionated regimens have become widely implemented in clinical practice and is recognized as a viable treatment approach in current guidelines [5, 6, 32]. More recently, aiming to improve patient compliance while reducing toxicity associated with radiotherapy, the SDR-RT trial [8] compared SDR-RT (a high-dose once-daily regimen delivering 50 Gy to the CTV and 60 Gy to the GTV) with conventional fractionated radiotherapy (CF-RT) (a high-dose once-daily regimen delivering 60 Gy to both CTV and GTV). It demonstrated that SDR-RT provided similar survival outcomes while offering better OAR-sparing and lower incidences of acute grade ≥ 3 toxicities and radiation pneumonitis (54.5% vs. 66.9%, *P* = 0.020; 2.4% vs. 7.7%, *P* = 0.027). Although the SDR-RT trial’s target volume delineations included high-risk uninvolved lymph nodes in the CTV, this was not reported in detail. In our study, we further observed that ENI in high-risk lymph node regions was associated with significantly improved OS, PFS, and DMFS, and with a lower cumulative incidence of ENF. These findings persisted after multivariable adjustment and PSM, supporting a potential role for ENI in contemporary radiotherapy. Furthermore, ENI was not more radiotoxic than IFRT in our study (Table 2), as IMRT and the SDR-RT strategy provided the technical basis for implementing ENI without increasing toxicity to normal tissues [8, 14, 33–35]. Meanwhile, enforcing stricter patient qualification criteria, particularly for lung function, and adhering to strict normal tissue dose limits are critical prerequisites for achieving this toxicity reduction (Table S5).

A few studies have directly compared survival outcomes between retaining ENI and omitting ENI in LS-SCLC. Graabak et al. [36] reported no significant difference in median overall survival (24 vs. 25 months, *P* = 0.59) or median progression-free survival (11 vs. 11 months, *P* = 0.23) for IFI based on PET-CT compared with ENI based on CT. However, in this study, the ENI range was confined to 4–7 lymph node stations and excluded some high-risk nodal regions as well as low-dose areas, such as the upper mediastinum and supraclavicular regions, thereby potentially limiting the efficacy of ENI. Across earlier studies from the 2D to IMRT era, ENI definitions have varied widely, from treating only mediastinal nodes to including mediastinal plus supraclavicular regions or a few selective nodal groups (4–7 mediastinal lymph node stations) [36–38]. Such inconsistency in target delineation makes direct comparison difficult and may explain the conflicting results across trials. Unlike the above ENI method, in our study, the ENI region was limited to high-risk, uninvolved lymph node stations, as determined by the location of the primary lesion, involved mediastinal lymph nodes, and lymphatic drainage patterns. Our study data indicate that the mean accidental dose of IFRT in high-risk lymph node regions is approximately 20 Gy, which is relatively insufficient. (Table S8). Consequently, this targeted and selective ENI approach reduced unnecessary exposure to normal tissues while maintaining comprehensive coverage of potential microscopic disease, showing significant improvements in OS, PFS, and DMFS compared with IFRT.

Our further subgroup analysis of OS and PFS (Fig. 2) in the IFRT and ENI groups suggests that ENI seems to be particularly important for patients with primary lesions classified as T4, N3. Previous studies have shown that patients with N2 and N3 have a high recurrence rate in the upper mediastinum and supraclavicular lymph nodes [39]. It is recommended to expand the upper boundary of the radiation field to cover possible hidden small lesions. The T4 classification indicates that tumors exhibit greater aggressiveness. Meanwhile, we found that ENI significantly enhanced treatment outcomes for patients with late TRT (thoracic radiotherapy initiated concurrently with or after the third cycle of chemotherapy) and patients who have not received cCRT. This indicates that ENI is an effective strategy for patients who are unable to tolerate chemotherapy or miss the optimal timing for radiotherapy [40], thereby maximizing their treatment benefits. Additionally, ENI provided significant OS and PFS benefits in patients who underwent PET-CT. These observations suggest that ENI may compensate for undetected micrometastases beyond the sensitivity of PET imaging [41].

Immunotherapy has emerged as an effective approach for controlling micrometastatic disease by enhancing systemic immune surveillance, thereby delaying recurrence and improving overall survival in LS-SCLC [42]. In this study, we preliminarily observed that ENI continued to improve OS compared with IFRT in cohorts receiving immunotherapy (HR, 0.379, [95% CI: 0.144–0.993]; *P* = 0.040). Among those treated with ICIs, only 3 of 53 patients (5.7%) in the IFRT group developed ENF, whereas none of the 24 patients in the ENI group did, suggesting a possible synergistic interaction between ENI and immunotherapy. Previous studies have suggested that radiotherapy may damage tumor-draining lymph nodes (dLN) [43], but in our study, the limited ENI delineation in high risk lymph node regions with a relatively lower irradiated dose did not significantly impair survival outcomes. However, the current data are not yet mature, the hypothesis warrants further investigation in prospective trials, especially given that existing relevant studies have not specified target volume definitions.

Rosenzweig’s definition of ENF was followed in our research. However, as his research worried, because the risk of in-field failure is higher, ENF will be covered up. This study employed a competitive risk model, using the first occurrence of death or primary tumor failure or IFNF without evidence of ENF as competitive risk to calculate the cumulative ENF rate (pre-PSM: Gray’s *p* = 0.0319; post-PSM: Gray’s *p* < 0.001), thereby enhancing the accuracy of ENF incidence. Additionally, since only 21.4% of patients after PSM in our study underwent PCI, we opted not to exclude patients with distant metastases as the initial failure site to mitigate the risk of underestimating the true incidence of ENF [44]. To our knowledge, no prior prospective studies have directly compared the incidence of ENF between ENI and non-ENI groups. Our findings indicate that ENI was significantly associated with a reduction in the incidence of ENF. In the past studies, we have found that supraclavicular node recurrence is significantly associated with distant metastasis, resulting in shortened survival [24]. Data also support the role of subclinical disease as a source of subsequent metastases [45]. This may explain why ENI can effectively extend DMFS and OS, and the high profitable rate of SCLC seems to make ENI more important in improving the prognosis of LS-SCLC.

## Limitations

Our research has the following limitations. Firstly, this study was retrospective, and although propensity score matching was applied, selection bias cannot be entirely excluded. Secondly, patients treated with proton therapy were not included. Given that proton therapy markedly reduces incidental irradiation to uninvolved lymph node regions, our findings may help guide target delineation strategies in this setting. Specifically, they suggest that when incidental dose is minimized, as in proton therapy, elective nodal coverage should be considered to compensate for the loss of unintentional irradiation. Furthermore, the number of patients receiving immunotherapy was limited, and the follow-up period was insufficient to fully assess long-term outcomes. Future prospective studies are warranted to define optimal ENI dose thresholds, refine elective nodal target delineation, and validate the potential synergy between ENI and immunotherapy.

## Conclusion

In conclusion, we found that ENI in high-risk lymph node stations did not significantly increase the adverse reaction rate compared to IFRT, while effectively prolonging OS, PFS, and DMFS, and reducing the risk of ENF in patients with LS-SCLC. This finding offers a novel optimized angle for thoracic radiotherapy in LS-SCLC.

## Electronic Supplementary Material

Below is the link to the electronic supplementary material.

Supplementary Material 1

## Data Availability

The datasets generated during and/or analyzed during the current study are available from the corresponding author on reasonable request.
