# Supplementary material for: Elective nodal irradiation of high-risk regions is superior to involved field radiotherapy for limited-stage small cell lung cancer: a propensity score-matched retrospective study
Source: Radiat Oncol. 2026 Apr 28;21:87. doi: 10.1186/s13014-026-02845-6 (PMC13267226; doi:10.1186/s13014-026-02845-6)
Supplement: Supplementary file 1 — Supplementary Material 1 [file 13014_2026_2845_MOESM1_ESM.docx]

# Supplementary Materials

[Supplementary Materials 1](#_Toc1096)

[Supplementary Tables 2](#_Toc14122)

[Table S1. Baseline characteristics between the IFRT and ENI groups before PSM. 2](#_Toc8943)

[Table S2. Univariate and multivariate Cox regression analyses for clinical endpoint before PSM. 6](#_Toc9938)

[Table S3. Univariate and multivariate Cox regression analyses for clinical endpoint after PSM. 1](#_Toc2116)5

[Table S4.](#_Toc5255) *[Sensitivity analysis](#_Toc5255)* [2](#_Toc5255)5

[Table S5. Comparison of dosimetric indexes between ENI and IFRT groups. 2](#_Toc13862)5

[Table S6. Disease recurrence pattern. 2](#_Toc13701)6

[Table S7. Patient characteristics for patients with ENF. 2](#_Toc9320)6

[Table S8. The accidental irradiation doses to mediastinal uninvolved lymph node stations in IFRT patients. 2](#_Toc942)8

[Supplementary Figures 2](#_Toc18747)8

[Figure S1. The ENI delineation of a patient. 2](#_Toc22756)8

[Figure S2. Lymph node station distribution in ENI.. 3](#_Toc10664)0

[Figure S3. OS, PFS, DMFS and LRFS between the IFRT and ENI groups before PSM 3](#_Toc5649)1

## Supplementary Tables

### **Table S1**. Baseline characteristics between the IFRT and ENI groups before PSM.

| **Characteristic** | **IFRT (n = 344)** | **ENI (n = 196)** | ***P-value*** |
| --- | --- | --- | --- |
| Gender, n (%) |  |  | 0.766 |
| Female | 90 (26.16) | 49 (25.00) |  |
| Male | 254 (73.84) | 147 (75.00) |  |
| Age, n (%) |  |  | 0.270 |
| >60 | 143 (41.57) | 72 (36.73) |  |
| ≤60 | 201 (58.43) | 124 (63.27) |  |
| ECOG, n (%) |  |  | 0.705 |
| 0 | 73 (21.22) | 41 (20.92) |  |
| 1 | 262 (76.16) | 152 (77.55) |  |
| 2 | 9 (2.62) | 3 (1.53) |  |
| T, n (%) |  |  | 0.750 |
| 1 | 67 (19.48) | 33 (16.84) |  |
| 2 | 116 (33.72) | 72 (36.73) |  |
| 3 | 65 (18.90) | 33 (16.84) |  |
| 4 | 96 (27.91) | 58 (29.59) |  |
| N, n (%) |  |  | 0.826 |
| 0 | 11 (3.20) | 5 (2.55) |  |
| 1 | 31 (9.01) | 18 (9.18) |  |
| 2 | 138 (40.12) | 86 (43.88) |  |
| 3 | 164 (47.67) | 87 (44.39) |  |
| Clinical Stage, n (%) |  |  | 0.222 |
| Ⅰ-Ⅱ | 17 (4.94) | 15 (7.65) |  |
| ⅢA | 74 (21.51) | 52 (26.53) |  |
| ⅢB | 181 (52.62) | 88 (44.90) |  |
| ⅢC | 72 (20.93) | 41 (20.92) |  |
| SCLN metastasis, n (%) |  |  | **<.001** |
| No | 235 (68.31) | 172 (87.76) |  |
| Bilateral metastasis | 41 (11.92) | 0 (0.00) |  |
| Unilateral metastasis | 68 (19.77) | 24 (12.24) |  |
| Tumor type, n (%) |  |  | **0.031** |
| Central | 218 (63.37) | 142 (72.45) |  |
| Peripheral | 126 (36.63) | 54 (27.55) |  |
| Smoking, n (%) |  |  | 0.428 |
| No | 95 (27.62) | 48 (24.49) |  |
| Yes | 249 (72.38) | 148 (75.51) |  |
| GTV volume (cc), n (%) |  |  | 0.107 |
| ＞46 | 181 (52.62) | 89 (45.41) |  |
| ≤46 | 163 (47.38) | 107 (54.59) |  |
| PCI, n (%) |  |  | 0.749 |
| No | 276 (80.23) | 155 (79.08) |  |
| Yes | 68 (19.77) | 41 (20.92) |  |
| Radiotherapy modality, n (%) |  |  | **<.001** |
| IMRT | 235 (68.31) | 100 (51.02) |  |
| VMAT | 109 (31.69) | 96 (48.98) |  |
| CCRT, n (%) |  |  | 0.852 |
| No | 199 (57.85) | 115 (58.67) |  |
| Yes | 145 (42.15) | 81 (41.33) |  |
| Combined with ICIs, n (%) |  |  | 0.312 |
| No | 291 (84.59) | 172 (87.76) |  |
| Yes | 53 (15.41) | 24 (12.24) |  |
| TTRT, n (%) |  |  | 0.070 |
| early TRT | 146 (42.44) | 99 (50.51) |  |
| late TRT | 198 (57.56) | 97 (49.49) |  |
| Total chemotherapy cycles, n (%) |  |  | 0.578 |
| 2-4 | 177 (51.45) | 110 (56.12) |  |
| 5-6 | 155 (45.06) | 80 (40.82) |  |
| >6 | 12 (3.49) | 6 (3.06) |  |
| Induction chemotherapy cycles, n (%) |  |  | 0.166 |
| 1-2 | 146 (42.44) | 99 (50.51) |  |
| 3-4 | 149 (43.31) | 70 (35.71) |  |
| ≥5 | 49 (14.24) | 27 (13.78) |  |
| Concurrent chemotherapy cycles, n (%) |  |  | 0.296 |
| 0 | 199 (57.85) | 115 (58.67) |  |
| 1 | 94 (27.33) | 44 (22.45) |  |
| 2 | 51 (14.83) | 37 (18.88) |  |
| Consolidation chemotherapy cycles, n (%) |  |  | 0.751 |
| 0 | 170 (49.42) | 93 (47.45) |  |
| 1-2 | 133 (38.66) | 82 (41.84) |  |
| ≥3 | 41 (11.92) | 21 (10.71) |  |
| PET CT, n (%) |  |  | 0.524 |
| No | 178 (51.74) | 107 (54.59) |  |
| Yes | 166 (48.26) | 89 (45.41) |  |

*Abbreviation: ECOG, Eastern Cooperative Oncology Group; SCLN, supraclavicular lymph node; PCI, Prophylactic Cranial Irradiation; CCRT, concurrent chemoradiotherapy; ICIs, immune checkpoint inhibitors; TTRT, the timing of thoracic radiotherapy. early TRT, thoracic radiotherapy initiated after 1-2 cycles of chemotherapy. late TRT, thoracic radiotherapy initiated after 3 or more cycles of chemotherapy; PET, positron emission tomography*

### **Table S2. Univariate and multivariate Cox regression analyses for clinical endpoint before PSM.**

| **Factor** | **Univariate** | | | | | | | | **Multivariate** | | | | | | | |
| --- | --- | --- | --- | --- | --- | --- | --- | --- | --- | --- | --- | --- | --- | --- | --- | --- |
|  | **OS** | | **PFS** | | **DMFS** | | **LRFS** | | **OS** | | **PFS** | | **DMFS** | | **LRFS** | |
|  | *P-value* | HR (95%CI) | *P-value* | HR (95%CI) | *P-value* | HR (95%CI) | *P-value* | HR (95%CI) | *P-value* | HR (95%CI) | *P-value* | HR (95%CI) | *P-value* | HR (95%CI) | *P-value* | HR (95%CI) |
| radiotherapy target-definition strategies |  |  |  |  |  |  |  |  |  |  |  |  |  |  |  |  |
| IFRT |  | 1.00 (Reference) |  | 1.00 (Reference) |  | 1.00 (Reference) |  | 1.00 (Reference) |  | 1.00 (Reference) |  |  |  | 1.00 (Reference) |  |  |
| ENI | **0.006** | 0.71 (0.56 ~ 0.91) | 0.120 | 0.85 (0.69 ~ 1.04) | **0.041** | 0.77 (0.60 ~ 0.99) | 0.659 | 0.94 (0.71 ~ 1.24) | **0.006** | 0.71 (0.56 ~ 0.91) |  |  | 0.092 | 0.81 (0.63 ~ 1.04) |  |  |
| Gender |  |  |  |  |  |  |  |  |  |  |  |  |  |  |  |  |
| Female |  | 1.00 (Reference) |  | 1.00 (Reference) |  | 1.00 (Reference) |  | 1.00 (Reference) |  | 1.00 (Reference) |  |  |  | 1.00 (Reference) |  |  |
| Male | **0.044** | 1.33 (1.01 ~ 1.74) | 0.147 | 1.18 (0.94 ~ 1.49) | 0.085 | 1.28 (0.97 ~ 1.69) | 0.874 | 0.98 (0.71 ~ 1.33) | **0.020** | 1.41 (1.05 ~ 1.88) |  |  | 0.265 | 1.18 (0.88 ~ 1.59) |  |  |
| Age |  |  |  |  |  |  |  |  |  |  |  |  |  |  |  |  |
| >60 |  | 1.00 (Reference) |  | 1.00 (Reference) |  | 1.00 (Reference) |  | 1.00 (Reference) |  | 1.00 (Reference) |  |  |  | 1.00 (Reference) |  |  |
| ≤60 | **0.049** | 0.79 (0.63 ~ 0.99) | 0.623 | 1.05 (0.86 ~ 1.28) | **0.048** | 1.28 (1.01 ~ 1.64) | 0.493 | 1.11 (0.83 ~ 1.47) | 0.369 | 0.90 (0.70 ~ 1.14) |  |  | **0.004** | 1.45 (1.13 ~ 1.87) |  |  |
| ECOG |  |  |  |  |  |  |  |  |  |  |  |  |  |  |  |  |
| 0 |  | 1.00 (Reference) |  | 1.00 (Reference) |  | 1.00 (Reference) |  | 1.00 (Reference) |  | 1.00 (Reference) |  |  |  |  |  |  |
| 1 | **0.046** | 1.36 (1.01 ~ 1.83) | 0.872 | 0.98 (0.77 ~ 1.25) | 0.288 | 0.86 (0.65 ~ 1.14) | 0.465 | 0.89 (0.64 ~ 1.23) | 0.076 | 1.32 (0.97 ~ 1.81) |  |  |  |  |  |  |
| 2 | 0.090 | 1.85 (0.91 ~ 3.75) | 0.721 | 1.13 (0.58 ~ 2.17) | 0.823 | 1.09 (0.50 ~ 2.38) | 0.741 | 1.17 (0.46 ~ 2.94) | **0.035** | 2.20 (1.06 ~ 4.58) |  |  |  |  |  |  |
| T |  |  |  |  |  |  |  |  |  |  |  |  |  |  |  |  |
| 1 |  | 1.00 (Reference) |  | 1.00 (Reference) |  | 1.00 (Reference) |  | 1.00 (Reference) |  | 1.00 (Reference) |  |  |  |  |  | 1.00 (Reference) |
| 2 | 0.279 | 1.21 (0.86 ~ 1.70) | 0.290 | 1.16 (0.88 ~ 1.54) | 0.402 | 1.15 (0.83 ~ 1.61) | 0.085 | 1.42 (0.95 ~ 2.13) | 0.099 | 1.34 (0.95 ~ 1.89) |  |  |  |  | **0.034** | 1.55 (1.03 ~ 2.33) |
| 3 | 0.458 | 1.16 (0.79 ~ 1.71) | 0.896 | 1.02 (0.74 ~ 1.42) | 0.732 | 0.93 (0.62 ~ 1.40) | 0.409 | 1.22 (0.76 ~ 1.95) | 0.453 | 1.17 (0.78 ~ 1.75) |  |  |  |  | 0.370 | 1.24 (0.77 ~ 2.00) |
| 4 | **0.016** | 1.53 (1.08 ~ 2.16) | 0.137 | 1.25 (0.93 ~ 1.66) | 0.138 | 1.30 (0.92 ~ 1.84) | 0.269 | 1.27 (0.83 ~ 1.95) | 0.050 | 1.43 (1.01 ~ 2.04) |  |  |  |  | 0.553 | 1.14 (0.74 ~ 1.77) |
| N |  |  |  |  |  |  |  |  |  |  |  |  |  |  |  |  |
| 0 |  | 1.00 (Reference) |  | 1.00 (Reference) |  | 1.00 (Reference) |  | 1.00 (Reference) |  |  |  |  |  |  |  |  |
| 1 | 0.302 | 0.68 (0.33 ~ 1.42) | 0.395 | 0.75 (0.39 ~ 1.45) | 0.436 | 0.71 (0.30 ~ 1.68) | 0.113 | 0.52 (0.23 ~ 1.17) |  |  |  |  |  |  |  |  |
| 2 | 0.367 | 0.74 (0.39 ~ 1.42) | 0.788 | 0.92 (0.51 ~ 1.66) | 0.879 | 1.06 (0.50 ~ 2.27) | 0.174 | 0.62 (0.31 ~ 1.24) |  |  |  |  |  |  |  |  |
| 3 | 0.955 | 1.02 (0.54 ~ 1.93) | 0.618 | 1.16 (0.65 ~ 2.08) | 0.590 | 1.23 (0.58 ~ 2.63) | 0.457 | 0.77 (0.39 ~ 1.53) |  |  |  |  |  |  |  |  |
| SCLN metastasis, n (%) |  |  |  |  |  |  |  |  |  |  |  |  |  |  |  |  |
| No |  | 1.00 (Reference) |  | 1.00 (Reference) |  | 1.00 (Reference) |  | 1.00 (Reference) |  |  |  |  |  |  |  |  |
| Bilateral metastasis | 0.339 | 1.23 (0.80 ~ 1.89) | 0.543 | 1.12 (0.77 ~ 1.62) | 0.807 | 1.06 (0.67 ~ 1.67) | 0.864 | 1.05 (0.61 ~ 1.81) |  |  |  |  |  |  |  |  |
| Unilateral metastasis | 0.415 | 1.13 (0.84 ~ 1.53) | 0.375 | 1.12 (0.87 ~ 1.46) | 0.619 | 1.08 (0.79 ~ 1.48) | 0.636 | 1.09 (0.76 ~ 1.58) |  |  |  |  |  |  |  |  |
| Clinical Stage |  |  |  |  |  |  |  |  |  |  |  |  |  |  |  |  |
| Ⅰ-Ⅱ |  | 1.00 (Reference) |  | 1.00 (Reference) |  | 1.00 (Reference) |  | 1.00 (Reference) |  | 1.00 (Reference) |  | 1.00 (Reference) |  | 1.00 (Reference) |  | 1.00 (Reference) |
| ⅢA | 0.361 | 1.32 (0.73 ~ 2.41) | 0.240 | 1.35 (0.82 ~ 2.21) | 0.944 | 1.02 (0.59 ~ 1.77) | 0.070 | 1.99 (0.94 ~ 4.18) | 0.642 | 1.15 (0.63 ~ 2.11) | 0.363 | 1.26 (0.77 ~ 2.07) | 0.905 | 0.97 (0.55 ~ 1.69) | 0.111 | 1.84 (0.87 ~ 3.88) |
| ⅢB | **0.032** | 1.86 (1.06 ~ 3.27) | **0.012** | 1.83 (1.14 ~ 2.92) | 0.159 | 1.45 (0.86 ~ 2.43) | **0.047** | 2.08 (1.01 ~ 4.26) | 0.358 | 1.31 (0.74 ~ 2.33) | 0.139 | 1.43 (0.89 ~ 2.30) | 0.601 | 1.15 (0.68 ~ 1.94) | 0.189 | 1.63 (0.79 ~ 3.38) |
| ⅢC | **0.010** | 2.19 (1.21 ~ 3.96) | **0.008** | 1.96 (1.19 ~ 3.22) | 0.061 | 1.69 (0.98 ~ 2.94) | 0.079 | 1.98 (0.92 ~ 4.23) | 0.224 | 1.47 (0.79 ~ 2.72) | 0.194 | 1.40 (0.84 ~ 2.33) | 0.450 | 1.25 (0.70 ~ 2.20) | 0.362 | 1.44 (0.66 ~ 3.17) |
| Tumor type |  |  |  |  |  |  |  |  |  |  |  |  |  |  |  |  |
| Central |  | 1.00 (Reference) |  | 1.00 (Reference) |  | 1.00 (Reference) |  | 1.00 (Reference) |  |  |  |  |  |  |  |  |
| Peripheral | 0.721 | 1.05 (0.82 ~ 1.33) | 0.576 | 1.06 (0.86 ~ 1.30) | 0.187 | 1.18 (0.92 ~ 1.50) | 0.718 | 1.05 (0.79 ~ 1.41) |  |  |  |  |  |  |  |  |
| Smoking |  |  |  |  |  |  |  |  |  |  |  |  |  |  |  |  |
| No |  | 1.00 (Reference) |  | 1.00 (Reference) |  | 1.00 (Reference) |  | 1.00 (Reference) |  |  |  |  |  | 1.00 (Reference) |  |  |
| Yes | 0.351 | 1.13 (0.87 ~ 1.47) | 0.289 | 1.13 (0.90 ~ 1.41) | 0.094 | 1.27 (0.96 ~ 1.68) | 0.401 | 1.15 (0.83 ~ 1.58) |  |  |  |  | 0.291 | 1.17 (0.88 ~ 1.56) |  |  |
| GTV volume (cc) |  |  |  |  |  |  |  |  |  |  |  |  |  |  |  |  |
| ＞46 |  | 1.00 (Reference) |  | 1.00 (Reference) |  | 1.00 (Reference) |  | 1.00 (Reference) |  | 1.00 (Reference) |  | 1.00 (Reference) |  | 1.00 (Reference) |  | 1.00 (Reference) |
| ≤46 | **<.001** | 0.63 (0.50 ~ 0.80) | **<.001** | 0.61 (0.50 ~ 0.75) | **<.001** | 0.57 (0.45 ~ 0.72) | **<.001** | 0.62 (0.47 ~ 0.82) | **0.017** | 0.74 (0.58 ~ 0.95) | **<.001** | 0.64 (0.52 ~ 0.78) | **<.001** | 0.63 (0.49 ~ 0.81) | **<.001** | 0.57 (0.42 ~ 0.77) |
| PCI |  |  |  |  |  |  |  |  |  |  |  |  |  |  |  |  |
| No |  | 1.00 (Reference) |  | 1.00 (Reference) |  | 1.00 (Reference) |  | 1.00 (Reference) |  | 1.00 (Reference) |  | 1.00 (Reference) |  | 1.00 (Reference) |  | 1.00 (Reference) |
| Yes | **<.001** | 0.49 (0.36 ~ 0.68) | **<.001** | 0.57 (0.44 ~ 0.73) | **<.001** | 0.54 (0.39 ~ 0.74) | **0.016** | 0.64 (0.45 ~ 0.92) | **0.002** | 0.59 (0.42 ~ 0.83) | **<.001** | 0.64 (0.49 ~ 0.83) | **<.001** | 0.56 (0.40 ~ 0.78) | 0.201 | 0.79 (0.54 ~ 1.14) |
| Radiotherapy modality |  |  |  |  |  |  |  |  |  |  |  |  |  |  |  |  |
| IMRT |  | 1.00 (Reference) |  | 1.00 (Reference) |  | 1.00 (Reference) |  | 1.00 (Reference) |  |  |  |  |  |  |  |  |
| VMAT | 0.957 | 1.01 (0.80 ~ 1.27) | 0.220 | 1.13 (0.93 ~ 1.38) | 0.324 | 1.13 (0.89 ~ 1.43) | 0.157 | 1.22 (0.93 ~ 1.61) |  |  |  |  |  |  |  |  |
| CCRT |  |  |  |  |  |  |  |  |  |  |  |  |  |  |  |  |
| No |  | 1.00 (Reference) |  | 1.00 (Reference) |  | 1.00 (Reference) |  | 1.00 (Reference) |  | 1.00 (Reference) |  | 1.00 (Reference) |  | 1.00 (Reference) |  | 1.00 (Reference) |
| Yes | **0.001** | 0.67 (0.53 ~ 0.85) | **<.001** | 0.71 (0.58 ~ 0.87) | **0.007** | 0.72 (0.56 ~ 0.91) | **<.001** | 0.59 (0.44 ~ 0.78) | **0.005** | 0.70 (0.54 ~ 0.90) | 0.067 | 0.82 (0.67 ~ 1.01) | 0.151 | 0.83 (0.64 ~ 1.07) | **0.002** | 0.61 (0.45 ~ 0.83) |
| Combined with ICIs |  |  |  |  |  |  |  |  |  |  |  |  |  |  |  |  |
| No |  | 1.00 (Reference) |  | 1.00 (Reference) |  | 1.00 (Reference) |  | 1.00 (Reference) |  | 1.00 (Reference) |  | 1.00 (Reference) |  | 1.00 (Reference) |  |  |
| Yes | **0.032** | 0.66 (0.45 ~ 0.97) | 0.077 | 0.77 (0.57 ~ 1.03) | **0.027** | 0.66 (0.45 ~ 0.95) | 0.351 | 0.82 (0.54 ~ 1.24) | **0.037** | 0.66 (0.45 ~ 0.98) | 0.077 | 0.76 (0.57 ~ 1.03) | **0.031** | 0.66 (0.45 ~ 0.96) |  |  |
| TTRT |  |  |  |  |  |  |  |  |  |  |  |  |  |  |  |  |
| early TRT |  | 1.00 (Reference) |  | 1.00 (Reference) |  | 1.00 (Reference) |  | 1.00 (Reference) |  | 1.00 (Reference) |  | 1.00 (Reference) |  | 1.00 (Reference) |  | 1.00 (Reference) |
| late TRT | 0.062 | 1.24 (0.99 ~ 1.57) | **0.009** | 1.30 (1.07 ~ 1.58) | **0.001** | 1.49 (1.17 ~ 1.89) | **0.025** | 1.38 (1.04 ~ 1.83) | 0.425 | 1.10 (0.87 ~ 1.40) | 0.072 | 1.21 (0.98 ~ 1.48) | **0.011** | 1.38 (1.08 ~ 1.77) | 0.283 | 1.17 (0.88 ~ 1.57) |
| Total chemotherapy cycles |  |  |  |  |  |  |  |  |  |  |  |  |  |  |  |  |
| 2-4 |  | 1.00 (Reference) |  | 1.00 (Reference) |  | 1.00 (Reference) |  | 1.00 (Reference) |  |  |  |  |  |  |  |  |
| 5-6 | 0.667 | 0.95 (0.75 ~ 1.20) | 0.460 | 0.93 (0.76 ~ 1.13) | 0.288 | 1.14 (0.90 ~ 1.44) | 0.613 | 1.08 (0.81 ~ 1.42) |  |  |  |  |  |  |  |  |
| >6 | 0.946 | 0.98 (0.52 ~ 1.85) | 0.759 | 1.09 (0.64 ~ 1.83) | 0.478 | 1.25 (0.68 ~ 2.31) | 0.280 | 1.46 (0.74 ~ 2.88) |  |  |  |  |  |  |  |  |
| PET CT |  |  |  |  |  |  |  |  |  |  |  |  |  |  |  |  |
| No |  | 1.00 (Reference) |  | 1.00 (Reference) |  | 1.00 (Reference) |  | 1.00 (Reference) |  |  |  |  |  |  |  |  |
| Yes | 0.923 | 1.01 (0.81 ~ 1.27) | 0.703 | 0.96 (0.79 ~ 1.17) | 0.904 | 0.99 (0.78 ~ 1.25) | 0.851 | 1.03 (0.78 ~ 1.35) |  |  |  |  |  |  |  |  |

*Abbreviation: ECOG, Eastern Cooperative Oncology Group; SCLN, supraclavicular lymph node; PCI, Prophylactic Cranial Irradiation; CCRT, concurrent chemoradiotherapy; ICIs, immune checkpoint inhibitors; TTRT, the timing of thoracic radiotherapy. early TRT, thoracic radiotherapy initiated after 1-2 cycles of chemotherapy. late TRT, thoracic radiotherapy initiated after 3 or more cycles of chemotherapy; PET, positron emission tomography*

### **Table S3. Univariate and multivariate Cox regression analyses for clinical endpoint after PSM.**

| **Factor** | **Univariate** | | | | | | | | **Multivariate** | | | | | | | |
| --- | --- | --- | --- | --- | --- | --- | --- | --- | --- | --- | --- | --- | --- | --- | --- | --- |
|  | **OS** | | **PFS** | | **DMFS** | | **LRFS** | | **OS** | | **PFS** | | **DMFS** | | **LRFS** | |
|  | ***P-value*** | **HR (95%CI)** | ***P-value*** | **HR (95%CI)** | ***P-value*** | **HR (95%CI)** | ***P-value*** | **HR (95%CI)** | ***P-value*** | **HR (95%CI)** | ***P-value*** | **HR (95%CI)** | ***P-value*** | **HR (95%CI)** | ***P-value*** | **HR (95%CI)** |
| radiotherapy target-definition strategies |  |  |  |  |  |  |  |  |  |  |  |  |  |  |  |  |
| IFRT |  | 1.00 (Reference) |  | 1.00 (Reference) |  | 1.00 (Reference) |  | 1.00 (Reference) |  | 1.00 (Reference) |  | 1.00 (Reference) |  |  |  | 1.00 (Reference) |
| ENI | **0.008** | 0.69 (0.52 ~ 0.90) | **0.007** | 0.72 (0.57 ~ 0.91) | **0.004** | 0.67 (0.50 ~ 0.88) | 0.109 | 0.77 (0.55 ~ 1.06) | **0.010** | 0.69 (0.52 ~ 0.92) | **0.002** | 0.69 (0.54 ~ 0.87) |  |  | **0.007** | 0.68 (0.51 ~ 0.90) |
| Gender |  |  |  |  |  |  |  |  |  |  |  |  |  |  |  |  |
| Female |  | 1.00 (Reference) |  | 1.00 (Reference) |  | 1.00 (Reference) |  | 1.00 (Reference) |  | 1.00 (Reference) |  |  |  | 1.00 (Reference) |  | 1.00 (Reference) |
| Male | **0.043** | 1.44 (1.01 ~ 2.04) | 0.103 | 1.26 (0.95 ~ 1.68) | 0.054 | 1.41 (0.99 ~ 1.99) | 0.961 | 1.01 (0.69 ~ 1.48) | **0.035** | 1.48 (1.03 ~ 2.12) |  |  | **0.010** | 0.69 (0.52 ~ 0.91) | 0.147 | 1.30 (0.91 ~ 1.86) |
| Age |  |  |  |  |  |  |  |  |  |  |  |  |  |  |  |  |
| >60 |  | 1.00 (Reference) |  | 1.00 (Reference) |  | 1.00 (Reference) |  | 1.00 (Reference) |  |  |  |  |  | 1.00 (Reference) |  | 1.00 (Reference) |
| ≤60 | 0.171 | 0.82 (0.62 ~ 1.09) | 0.556 | 1.07 (0.85 ~ 1.37) | **0.048** | 1.35 (1.01 ~ 1.82) | 0.830 | 1.04 (0.74 ~ 1.45) |  |  |  |  | **0.012** | 1.57 (1.10 ~ 2.25) | **0.005** | 1.55 (1.14 ~ 2.12) |
| ECOG |  |  |  |  |  |  |  |  |  |  |  |  |  |  |  |  |
| 0 |  | 1.00 (Reference) |  | 1.00 (Reference) |  | 1.00 (Reference) |  | 1.00 (Reference) |  | 1.00 (Reference) |  |  |  | 1.00 (Reference) |  |  |
| 1 | **0.016** | 1.60 (1.09 ~ 2.35) | 0.594 | 1.08 (0.81 ~ 1.46) | 0.924 | 0.98 (0.70 ~ 1.38) | 0.931 | 0.98 (0.66 ~ 1.46) | **0.019** | 1.59 (1.08 ~ 2.35) |  |  | 0.155 | 0.80 (0.59 ~ 1.09) |  |  |
| 2 | 0.059 | 2.48 (0.96 ~ 6.40) | 0.312 | 1.55 (0.66 ~ 3.60) | 0.100 | 2.05 (0.87 ~ 4.83) | 0.111 | 2.34 (0.82 ~ 6.63) | 0.065 | 2.46 (0.95 ~ 6.39) |  |  |  |  |  |  |
| T |  |  |  |  |  |  |  |  |  |  |  |  |  |  |  |  |
| 1 |  | 1.00 (Reference) |  | 1.00 (Reference) |  | 1.00 (Reference) |  | 1.00 (Reference) |  |  |  |  |  |  |  | 1.00 (Reference) |
| 2 | 0.685 | 1.09 (0.72 ~ 1.64) | 0.440 | 1.14 (0.81 ~ 1.61) | 0.511 | 1.15 (0.76 ~ 1.73) | 0.213 | 1.37 (0.84 ~ 2.23) |  |  |  |  |  |  | 0.390 | 1.20 (0.79 ~ 1.82) |
| 3 | 0.939 | 0.98 (0.61 ~ 1.58) | 0.840 | 0.96 (0.64 ~ 1.44) | 0.748 | 0.92 (0.56 ~ 1.51) | 0.723 | 1.11 (0.63 ~ 1.97) |  |  |  |  |  | 1.00 (Reference) | 0.626 | 0.88 (0.54 ~ 1.45) |
| 4 | 0.204 | 1.32 (0.86 ~ 2.01) | 0.157 | 1.29 (0.91 ~ 1.85) | 0.091 | 1.44 (0.94 ~ 2.19) | 0.330 | 1.29 (0.77 ~ 2.17) |  |  |  |  | 0.074 | 1.31 (0.97 ~ 1.75) | 0.487 | 1.16 (0.76 ~ 1.79) |
| N |  |  |  |  |  |  |  |  |  |  |  |  |  |  |  |  |
| 0 |  | 1.00 (Reference) |  | 1.00 (Reference) |  | 1.00 (Reference) |  | 1.00 (Reference) |  |  |  |  |  |  |  |  |
| 1 | 0.253 | 0.60 (0.25 ~ 1.44) | 0.404 | 0.72 (0.34 ~ 1.55) | 0.504 | 0.71 (0.26 ~ 1.95) | 0.184 | 0.54 (0.22 ~ 1.34) |  |  |  |  |  |  |  |  |
| 2 | 0.224 | 0.62 (0.28 ~ 1.34) | 0.672 | 0.86 (0.44 ~ 1.70) | 0.897 | 1.06 (0.43 ~ 2.62) | 0.100 | 0.52 (0.24 ~ 1.13) |  |  |  |  |  |  |  |  |
| 3 | 0.937 | 0.97 (0.45 ~ 2.09) | 0.673 | 1.16 (0.59 ~ 2.28) | 0.503 | 1.36 (0.55 ~ 3.35) | 0.511 | 0.77 (0.35 ~ 1.68) |  |  |  |  |  |  |  |  |
| SCLN metastasis, n (%) |  |  |  |  |  |  |  |  |  |  |  |  |  |  |  |  |
| No |  | 1.00 (Reference) |  | 1.00 (Reference) |  | 1.00 (Reference) |  | 1.00 (Reference) |  |  |  | 1.00 (Reference) |  |  |  | 1.00 (Reference) |
| Unilateral metastasis | 0.149 | 1.32 (0.91 ~ 1.93) | 0.078 | 1.34 (0.97 ~ 1.85) | **0.032** | 1.49 (1.04 ~ 2.15) | 0.160 | 1.37 (0.88 ~ 2.12) |  |  | 0.247 | 1.22 (0.87 ~ 1.72) |  |  | 0.284 | 1.23 (0.84 ~ 1.79) |
| Clinical Stage |  |  |  |  |  |  |  |  |  |  |  |  |  |  |  |  |
| Ⅰ-Ⅱ |  | 1.00 (Reference) |  | 1.00 (Reference) |  | 1.00 (Reference) |  | 1.00 (Reference) |  | 1.00 (Reference) |  | 1.00 (Reference) |  |  |  |  |
| ⅢA | 0.464 | 1.27 (0.67 ~ 2.39) | 0.324 | 1.30 (0.77 ~ 2.18) | 0.680 | 0.89 (0.50 ~ 1.57) | 0.081 | 2.04 (0.92 ~ 4.55) | 0.675 | 1.15 (0.60 ~ 2.18) | 0.360 | 1.28 (0.76 ~ 2.16) |  |  |  |  |
| ⅢB | **0.021** | 2.02 (1.11 ~ 3.68) | **0.004** | 2.08 (1.27 ~ 3.39) | 0.118 | 1.52 (0.90 ~ 2.58) | **0.017** | 2.58 (1.19 ~ 5.60) | 0.187 | 1.51 (0.82 ~ 2.80) | **0.034** | 1.72 (1.04 ~ 2.85) |  |  |  |  |
| ⅢC | 0.051 | 1.90 (1.00 ~ 3.64) | **0.047** | 1.73 (1.01 ~ 2.96) | 0.196 | 1.47 (0.82 ~ 2.63) | 0.097 | 2.03 (0.88 ~ 4.70) | 0.320 | 1.40 (0.72 ~ 2.74) | 0.478 | 1.23 (0.70 ~ 2.16) |  |  |  |  |
| Tumor type |  |  |  |  |  |  |  |  |  |  |  |  |  |  |  |  |
| Central |  | 1.00 (Reference) |  | 1.00 (Reference) |  | 1.00 (Reference) |  | 1.00 (Reference) |  |  |  |  |  |  |  |  |
| Peripheral | 0.903 | 1.02 (0.75 ~ 1.38) | 0.487 | 1.09 (0.85 ~ 1.41) | 0.302 | 1.17 (0.87 ~ 1.57) | 0.950 | 1.01 (0.71 ~ 1.44) |  |  |  |  |  |  |  |  |
| Smoking |  |  |  |  |  |  |  |  |  |  |  |  |  |  |  |  |
| No |  | 1.00 (Reference) |  | 1.00 (Reference) |  | 1.00 (Reference) |  | 1.00 (Reference) |  |  |  |  |  |  |  |  |
| Yes | 0.158 | 1.27 (0.91 ~ 1.77) | 0.285 | 1.16 (0.88 ~ 1.53) | 0.161 | 1.28 (0.91 ~ 1.79) | 0.290 | 1.24 (0.83 ~ 1.84) |  |  |  |  |  |  |  |  |
| GTV volume (cc) |  |  |  |  |  |  |  |  |  |  |  |  |  |  |  |  |
| ＞46 |  | 1.00 (Reference) |  | 1.00 (Reference) |  | 1.00 (Reference) |  | 1.00 (Reference) |  | 1.00 (Reference) |  | 1.00 (Reference) |  |  |  | 1.00 (Reference) |
| ≤46 | **0.001** | 0.64 (0.48 ~ 0.84) | **<.001** | 0.57 (0.45 ~ 0.73) | **<.001** | 0.55 (0.42 ~ 0.73) | **0.008** | 0.64 (0.47 ~ 0.89) | 0.050 | 0.74 (0.55 ~ 0.99) | **<.001** | 0.60 (0.47 ~ 0.77) |  |  | **<.001** | 0.58 (0.43 ~ 0.79) |
| PCI |  |  |  |  |  |  |  |  |  |  |  |  |  |  |  |  |
| No |  | 1.00 (Reference) |  | 1.00 (Reference) |  | 1.00 (Reference) |  | 1.00 (Reference) |  | 1.00 (Reference) |  | 1.00 (Reference) |  |  |  | 1.00 (Reference) |
| Yes | **0.002** | 0.54 (0.37 ~ 0.79) | **0.001** | 0.60 (0.44 ~ 0.82) | **<.001** | 0.49 (0.33 ~ 0.72) | 0.213 | 0.77 (0.51 ~ 1.16) | 0.098 | 0.71 (0.47 ~ 1.07) | 0.083 | 0.75 (0.54 ~ 1.04) |  |  | **0.003** | 0.53 (0.35 ~ 0.81) |
| Radiotherapy modality |  |  |  |  |  |  |  |  |  |  |  |  |  |  |  |  |
| IMRT |  | 1.00 (Reference) |  | 1.00 (Reference) |  | 1.00 (Reference) |  | 1.00 (Reference) |  |  |  |  |  | 1.00 (Reference) |  |  |
| VMAT | 0.292 | 1.16 (0.88 ~ 1.54) | 0.152 | 1.19 (0.94 ~ 1.50) | 0.104 | 1.26 (0.95 ~ 1.66) | 0.211 | 1.23 (0.89 ~ 1.70) |  |  |  |  | 0.914 | 0.96 (0.49 ~ 1.89) |  |  |
| CCRT |  |  |  |  |  |  |  |  |  |  |  |  | 0.438 | 1.29 (0.68 ~ 2.43) |  |  |
| No |  | 1.00 (Reference) |  | 1.00 (Reference) |  | 1.00 (Reference) |  | 1.00 (Reference) |  | 1.00 (Reference) |  | 1.00 (Reference) | 0.260 | 1.47 (0.75 ~ 2.87) |  | 1.00 (Reference) |
| Yes | **0.005** | 0.67 (0.50 ~ 0.88) | **0.006** | 0.72 (0.57 ~ 0.91) | **0.014** | 0.70 (0.53 ~ 0.93) | **0.006** | 0.63 (0.45 ~ 0.87) | **0.012** | 0.68 (0.50 ~ 0.92) | 0.124 | 0.82 (0.63 ~ 1.06) |  |  | 0.162 | 0.80 (0.59 ~ 1.09) |
| Combined with ICIs |  |  |  |  |  |  |  |  |  |  |  |  |  |  |  |  |
| No |  | 1.00 (Reference) |  | 1.00 (Reference) |  | 1.00 (Reference) |  | 1.00 (Reference) |  | 1.00 (Reference) |  |  |  |  |  | 1.00 (Reference) |
| Yes | **0.006** | 0.46 (0.26 ~ 0.80) | 0.119 | 0.74 (0.51 ~ 1.08) | **0.034** | 0.59 (0.36 ~ 0.96) | 0.652 | 0.89 (0.54 ~ 1.46) | **0.015** | 0.49 (0.28 ~ 0.87) |  |  |  |  | 0.065 | 0.63 (0.39 ~ 1.03) |
| TTRT |  |  |  |  |  |  |  |  |  |  |  |  |  |  |  |  |
| early TRT |  | 1.00 (Reference) |  | 1.00 (Reference) |  | 1.00 (Reference) |  | 1.00 (Reference) |  |  |  | 1.00 (Reference) |  |  |  | 1.00 (Reference) |
| late TRT | 0.206 | 1.19 (0.91 ~ 1.57) | **0.005** | 1.40 (1.11 ~ 1.77) | **0.001** | 1.59 (1.20 ~ 2.11) | **0.010** | 1.54 (1.11 ~ 2.15) |  |  | **0.022** | 1.34 (1.04 ~ 1.72) |  | 1.00 (Reference) | **0.009** | 1.48 (1.10 ~ 1.98) |
| Total chemotherapy cycles |  |  |  |  |  |  |  |  |  |  |  |  | **0.009** | 0.59 (0.40 ~ 0.87) |  |  |
| 2-4 |  | 1.00 (Reference) |  | 1.00 (Reference) |  | 1.00 (Reference) |  | 1.00 (Reference) |  |  |  |  |  |  |  |  |
| 5-6 | 0.729 | 1.05 (0.79 ~ 1.39) | 0.947 | 1.01 (0.79 ~ 1.28) | 0.259 | 1.18 (0.89 ~ 1.56) | 0.143 | 1.28 (0.92 ~ 1.78) |  |  |  |  |  |  |  |  |
| >6 | 0.466 | 1.31 (0.64 ~ 2.68) | 0.866 | 1.06 (0.56 ~ 2.01) | 0.942 | 0.97 (0.43 ~ 2.21) | 0.255 | 1.62 (0.70 ~ 3.74) |  |  |  |  |  |  |  |  |
| PET CT |  |  |  |  |  |  |  |  |  |  |  |  |  |  |  |  |
| No |  | 1.00 (Reference) |  | 1.00 (Reference) |  | 1.00 (Reference) |  | 1.00 (Reference) |  |  |  |  |  |  |  |  |
| Yes | 0.322 | 1.15 (0.87 ~ 1.51) | 0.517 | 1.08 (0.86 ~ 1.36) | 0.754 | 1.05 (0.79 ~ 1.38) | 0.416 | 1.14 (0.83 ~ 1.58) |  |  |  |  |  |  |  |  |

*Abbreviation: ECOG, Eastern Cooperative Oncology Group; SCLN, supraclavicular lymph node; PCI, Prophylactic Cranial Irradiation; CCRT, concurrent chemoradiotherapy; ICIs, immune checkpoint inhibitors; TTRT, the timing of thoracic radiotherapy. early TRT, thoracic radiotherapy initiated after 1-2 cycles of chemotherapy. late TRT, thoracic radiotherapy initiated after 3 or more cycles of chemotherapy; PET, positron emission tomograph*

### **Table S4. Sensitivity analysis**

To assess the robustness of the results, two sensitivity analysis methods were used to control for confounding: multivariate Cox regression adjusted for the inverse probability of treatment weighting (IPTW) and standardized mortality ratio (SMR) weighting.

| Analysis | Clinical endpoint | HR | 95% CI | *P* |
| --- | --- | --- | --- | --- |
| Multivariate analysis with IPTW | OS | 0.70 | (0.55 - 0.90) | 0.006 |
|  | PFS | 0.81 | (0.66 - 1.00) | 0.049 |
|  | DMFS | 0.74 | (0.57 - 0.95) | 0.019 |
|  | LRFS | 0.90 | (0.67 - 1.20) | 0.463 |
| Multivariate analysis with SMR weighting | OS | 0.70 | (0.54 - 0.90) | 0.006 |
|  | PFS | 0.79 | (0.64 - 0.98) | 0.031 |
|  | DMFS | 0.71 | (0.55 - 0.92) | 0.011 |
|  | LRFS | 0.85 | (0.63 - 1.15) | 0.297 |

*Abbreviations: IPTW inverse probability of treatment weighting, SMR standardized mortality ratio OS overall survival, PFS progression-free survival, DMFS distant metastasis-free survival, LRFS locoregional recurrence-free survival, HR hazard ratio, CI confidence interval.*

### Table S5. Comparison of dosimetric indexes between ENI and IFRT groups.

| Target volume | DVH parameters | IFRT | ENI | *P* |
| --- | --- | --- | --- | --- |
|  |  | Mean ± SD | |  |
| PTV | D95 (Gy) | 53.51 ± 1.55 | 53.73 ± 1.14 | 0.360 |
|  | Mean dose (Gy) | 58.98 ± 1.32 | 58.80 ± 0.90 | 0.386 |
|  | Minimum dose (Gy) | 38.24 ± 8.51 | 39.62 ± 6.90 | 0.303 |
|  | Maximum dose (Gy) | 66.74 ± 2.05 | 66.62 ± 1.77 | 0.730 |
| PGTV | D95 (Gy) | 60.07 ± 1.49 | 60.43 ± 0.96 | 0.108 |
|  | Mean dose (Gy) | 62.40 ± 1.53 | 62.64 ± 0.95 | 0.278 |
|  | Minimum dose (Gy) | 55.13 ± 4.53 | 56.67 ± 3.24 | 0.028 |
|  | Maximum dose (Gy) | 66.70 ± 2.02 | 66.57 ± 1.78 | 0.698 |
| Lung | Mean dose (Gy) | 13.55 ± 1.50 | 13.35 ± 1.67 | 0.458 |
|  | V20 (%) | 24.43 ± 3.13 | 24.59 ± 3.21 | 0.762 |
| Heart | Mean dose (Gy) | 14.14 ± 6.21 | 13.00 ± 5.70 | 0.263 |
|  | V30 (%) | 18.96 ± 11.12 | 16.82 ± 9.48 | 0.228 |
|  | V40 (%) | 12.26 ± 7.92 | 10.62 ± 6.79 | 0.196 |
| Esophagus | Maximum dose (Gy) | 61.39 ± 3.44 | 60.19 ± 4.02 | 0.264 |
|  | Mean dose (Gy) | 23.11 ± 6.72 | 21.80 ± 7.84 | 0.528 |
| Cord | Maximum dose (Gy) | 35.31 ± 6.58 | 32.02 ± 7.40 | 0.132 |

### **Table S6. Disease recurrence pattern.**

| First site of failure (330failures among 540 patients) | N (%) |
| --- | --- |
| Distant metastasis only | 159(29.4) |
| Primary tumor failure and/or IFNF, without distant metastasis | 72(13.3) |
| Primary tumor failure and/or IFNF, combined with distant metastasis | 41(7.6) |
| Any Elective nodal failures (isolated and non-isolated) | 58(10.7) |
| Isolated Elective nodal failure | 27 |
| Non-isolated Elective nodal failure | 31 |

### **Table S7. Patient char**acteristics for patients with ENF.

| Patient with ENF | Treatment | ENF location (lymph node station) | Dose of ENF (Gy) | ENF within the elective irradiation region |
| --- | --- | --- | --- | --- |
| 1 | IFRT | 7 | 26.8 |  |
| 2 | IFRT | 1L | 3 |  |
| 3 | IFRT | 1L;1R | 2.1;2.0 |  |
| 4 | IFRT | 1R | 3.2 |  |
| 5 | IFRT | 6 | 25.6 |  |
| 6 | IFRT | 1R | 1.9 |  |
| 7 | IFRT | 1R | 2 |  |
| 8 | ENI | 1R | 1.9 |  |
| 9 | ENI | 1;3A | 3.6；42.6 |  |
| 10 | IFRT | 2R | 4.1 |  |
| 11 | IFRT | 1R;2R;6 | 1.4；15.6；35.9 |  |
| 12 | IFRT | 10L | 10 |  |
| 13 | IFRT | 3A;5;6 | 16.8；37.1；38 |  |
| 14 | IFRT | 6 | 26.9 |  |
| 15 | IFRT | 1L;1R;6 | 1；0.9;9.2 |  |
| 16 | IFRT | 2R;4R;6 | 11.2；8.4；16.7 |  |
| 17 | ENI | 10L | 8 |  |
| 18 | IFRT | 3A | 26.3 |  |
| 19 | IFRT | 1L;1R | 3.1;2.9 |  |
| 20 | ENI | 1L;2L | 52.8;54.2 | 1L;2L |
| 21 | IFRT | 1L;1R;3A;10R | 7.1；7.3;40.7；17.8 |  |
| 22 | IFRT | 1R | 3 |  |
| 23 | IFRT | 6 | 4.6 |  |
| 24 | IFRT | 2R | 29.5 |  |
| 25 | IFRT | 5;10L | 11.3；10.5 |  |
| 26 | IFRT | 1R | 3 |  |
| 27 | IFRT | 1R | 7.8 |  |
| 28 | IFRT | 1L | 2.4 |  |
| 29 | IFRT | 6 | 45.6 |  |
| 30 | ENI | 3A;6;7 | 40.6；7.2;54.4 | 7 |
| 31 | IFRT | 5 | 12.4 |  |
| 32 | IFRT | 1L;1R | 3；4.2 |  |
| 33 | IFRT | 4;7 | 30.5；11.2 |  |
| 34 | IFRT | 1R | 11.3 |  |
| 35 | IFRT | 3A;7 | 11.2；41.7 |  |
| 36 | IFRT | 1L;10L | 1；13 |  |
| 37 | IFRT | 5 | 3.8 |  |
| 38 | IFRT | 6 | 33.1 |  |
| 39 | ENI | 6 | 8.5 |  |
| 40 | IFRT | 2R;10R | 2；16 |  |
| 41 | IFRT | 1L | 9.5 |  |
| 42 | IFRT | 3P | 19.9；17 |  |
| 43 | IFRT | 1L;2L | 1；5 |  |
| 44 | ENI | 10R | 20.5 |  |
| 45 | IFRT | 8 | 18.4 |  |
| 46 | ENI | 3A;5;6 | 20.8；16.2；13 |  |
| 47 | ENI | 1L;1R | 1.7；1.5 |  |
| 48 | ENI | 4R;8 | 29;53.9 | 4R |
| 49 | IFRT | 10L | 34.8 |  |
| 50 | IFRT | 1L;1R;3A | 5；4；9.8 |  |
| 51 | IFRT | 10R | 28.1 |  |
| 52 | IFRT | 6 | 43.9 |  |
| 53 | ENI | 1L;1R | 5.2；4 |  |
| 54 | ENI | 1R | 7.5 |  |
| 55 | IFRT | 10L | 15.2 |  |
| 56 | ENI | 3A;6 | 27.7；10.6 |  |
| 57 | IFRT | 1L;1R | 28.8；9 |  |
| 58 | ENI | 3A | 23.4 |  |

*Abbreviation: Dose of ENF, for patients developed ENF, the recurrent site was registered on the original treatment planning CT, with the intersection point of the longest and shortest diameters of the recurrent area marked as the reference center point. The dose received at the center point was calculated to determine the dose received at the recurrent lymph node area.*

### **Table S8.** The accidental irradiation doses to mediastinal uninvolved lymph node stations in IFRT patients.

| lymph node station | mean dose (Gy) | |
| --- | --- | --- |
|  | Mean ± SD | Range |
| Total | 28.26 ± 18.02 | 0.34-59.96 |
| 1L | 13.12 ± 17.15 | 0.63-59.96 |
| 1R | 11.05 ± 16.44 | 0.34-55.97 |
| 2L | 25.51 ± 19.12 | 0.52-57.44 |
| 2R | 22.84 ± 18.18 | 1.43-58.57 |
| 3A | 34.20 ± 14.16 | 5.1-58.25 |
| 3P | 38.89 ± 15.20 | 2.00-59.67 |
| 4L | 36.22 ± 12.13 | 6.72-57.21 |
| 4R | 35.65 ± 12.82 | 13.08-50.21 |
| 5 | 32.71 ± 17.71 | 4.16-58.86 |
| 6 | 33.91 ± 11.62 | 8.92-55.34 |
| 7 | 44.43 ± 14.81 | 5.42-58.34 |
| 10L | 11.44 ± 5.69 | 1.54-35.63 |
| 10R | 18.93 ± 12.20 | 1.20-56.57 |
| High-risk uninvolved lymph node stations | 20.04 ± 16.73 | 0.46-59.96 |

## Supplementary Figures

### **Figure S1.**

The ENI delineation of a patient.The patient’s primary tumor was located in the right middle lobe, with pre-chemotherapy lymph node metastases to mediastinal stations 2, 3A, 4, 7, 8, and 10R. The clinical target volume (CTV) encompassed the post-chemotherapy residual primary tumor (GTV-T) with a 0.5 cm margin, included all pre-chemotherapy positive nodal regions, and delineated the nodal station 1L and 1R for elective nodal irradiation (ENI).


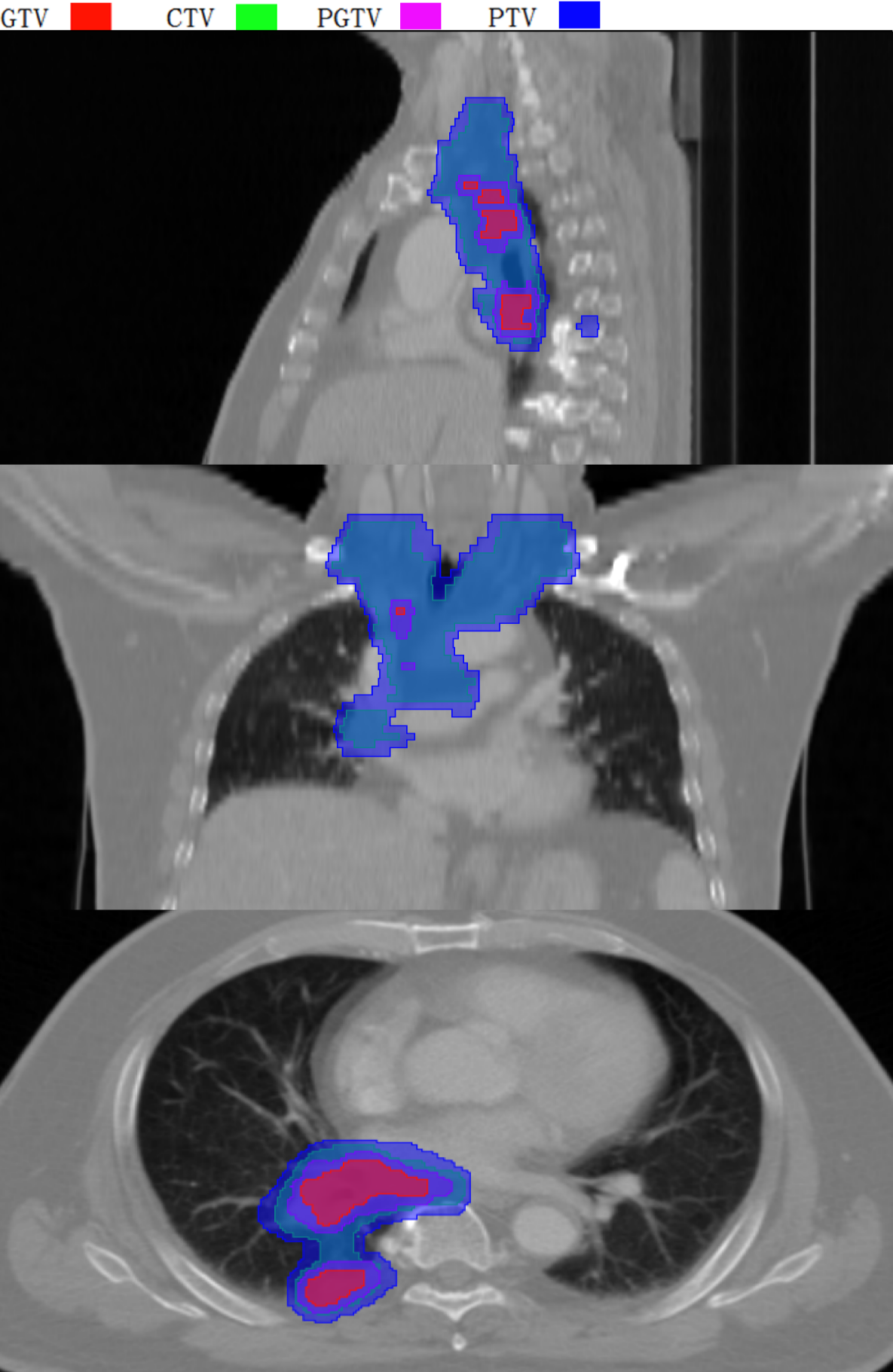


### **Figure S2.**

Lymph node station distribution in ENI. Among 196 patients who underwent ENI, a total of 326 lymph node stations selectively received ENI.

*
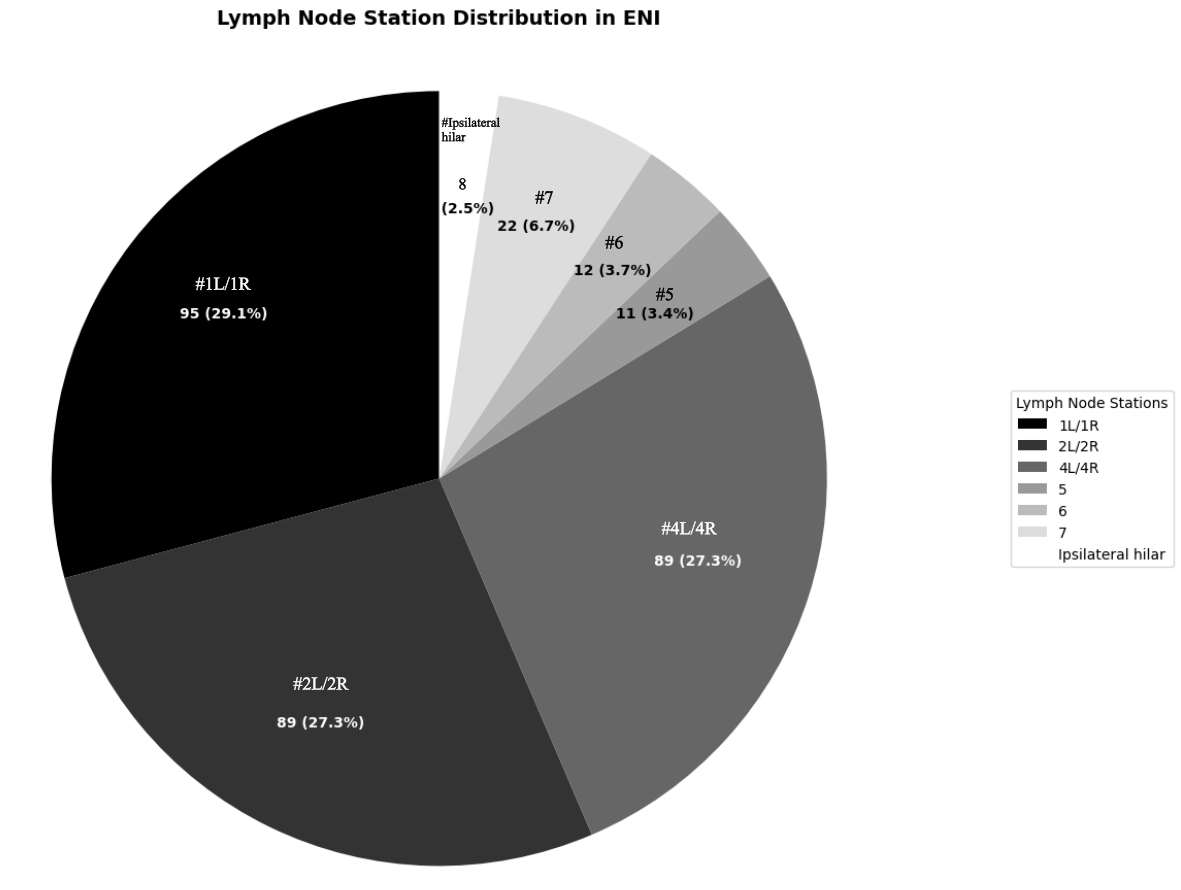
*

### **Figure S3.**

OS, PFS, DMFS and LRFS between the IFRT and ENI groups before PSM. (A) OS from the initiation of radiotherapy before PSM. (B) PFS from the initiation of radiotherapy before PSM. (C) DMFS from the initiation of radiotherapy before PSM. (D) LRFS from the initiation of radiotherapy before PSM.





*Abbreviation: OS overall survival, PFS progression-free survival, LRFS locoregional recurrence-free survival, IFRT Involved-field radiotherapy, DMFS distant metastasis-free survival; ENI, Elective Nodal Irradiation, PSM propensity score matching*
